# Incorporation and antimicrobial activity of nisin Z within carrageenan/chitosan multilayers

**DOI:** 10.1038/s41598-020-79702-3

**Published:** 2021-01-18

**Authors:** Jessie L. Webber, Rashin Namivandi-Zangeneh, Sławomir Drozdek, Kazimiera A. Wilk, Cyrille Boyer, Edgar H. H. Wong, Bronwyn H. Bradshaw-Hajek, Marta Krasowska, David A. Beattie

**Affiliations:** 1 Future Industries Institute, University of South Australia, Mawson Lakes, SA 5095 Australia; 2 UniSA STEM, University of South Australia, Mawson Lakes, SA 5095 Australia; 3 Centre for Advanced Macromolecular Design and Australian Centre for NanoMedicine, School of Chemical Engineering, University of New South Wales, Sydney, NSW 2052 Australia; 4 Department of Engineering and Technology of Chemical Processes, Faculty of Chemistry, Wrocław University of Science and Technology, Wybrzeże Wyspiańskiego 27, 50-370 Wrocław, Poland

**Keywords:** Antimicrobials, Polymers, Biosurfaces, Surface spectroscopy

## Abstract

An antimicrobial peptide, nisin Z, was embedded within polyelectrolyte multilayers (PEMs) composed of natural polysaccharides in order to explore the potential of forming a multilayer with antimicrobial properties. Using attenuated total reflection Fourier transform infrared spectroscopy (ATR FTIR), the formation of carrageenan/chitosan multilayers and the inclusion of nisin Z in two different configurations was investigated. Approximately 0.89 µg cm^−2^ nisin Z was contained within a 4.5 bilayer film. The antimicrobial properties of these films were also investigated. The peptide containing films were able to kill over 90% and 99% of planktonic and biofilm cells, respectively, against *Staphylococcus aureus* and methicillin-resistant *Staphylococcus aureus* (MRSA) strains compared to control films. Additionally, surface topography and wettability studies using atomic force microscopy (AFM) and the captive bubble technique revealed that surface roughness and hydrophobicity was similar for both nisin containing multilayers. This suggests that the antimicrobial efficacy of the peptide is unaffected by its location within the multilayer. Overall, these results demonstrate the potential to embed and protect natural antimicrobials within a multilayer to create functionalised coatings that may be desired by industry, such as in the food, biomaterials, and pharmaceutical industry sectors.

## Introduction

The prevention of microbial growth in the food, biomedical, and pharmaceutical industries is important for several reasons. Besides the physical instability and reduction of shelf life these microbes can cause in food-based products, foodborne illnesses and food poisoning are a major concern for consumers. These diseases are often caused by food contamination with bacteria including *Staphylococcus aureus* and *Listeria monocytogenes*^1,2^*.* In addition, minimising microbial growth in the biomedical scene can improve healing times and help to prevent infection. Consumer preference for natural products over synthetic chemical preservatives has driven current research in the direction of natural alternatives^3^. However, the efficiency of such molecules can be limited, due to a loss of antimicrobial activity arising from their direct dilution in food products or biological media, or from their interaction with other molecules and the surrounding environment^4^.

The incorporation of bioactive molecules into multilayers is a broadly studied topic and a well-established means to prevent this loss of antimicrobial activity, with a variety of biomolecules including proteins and peptides^5^, or particles^6,7^ and other small molecules^8^ able to be embedded into multilayers. Such films are already investigated for a variety of applications including for medical research as a means to accelerate wound healing^9^ or to lower infection rates due to biofilm growth on biomedical stents and other implanted devices^10^. In addition, the incorporation of natural antimicrobials into films for food processing equipment^10^ and for food packaging^11^, as well as edible and biodegradable food coatings are also becoming more commonplace, with the goal to prevent degradation of food products due to microbial spoilage^6^.

Antimicrobial peptides (AMPs), also termed host defence peptides, form part of the innate immune system of single celled microorganisms, insects and invertebrates, plants, fish, amphibians, birds and mammals, including humans. AMPs are typically made up of anywhere from 10 to 100 amino acids, are generally positively charged and are amphiphilic. While a generic mechanism of action for AMPs has not been conclusively elucidated^12–15^, AMPs have shown antimicrobial activity against a range of microbes including viruses, fungi and both Gram-positive and Gram-negative bacteria^16,17^. Despite high antimicrobial activity, most AMPs are not widely used in a clinical setting, due to limitations arising from toxicity and haemolytic activity within the body^12,18^, though there is a significant effort to find AMPs that are safe for clinical use^19^.

A number of different strategies have been explored to incorporate a range of bioactive AMPs into multilayers including direct deposition of AMPs during multilayer formation^5^, or by diffusion of AMPs into multilayers post formation^9^. Another method involves complexing the AMP with a polyelectrolyte used for film formation^20,21^. For example, a hydrophobic AMP can be complexed with a polyelectrolyte to incorporate it into a thin film^22^. Antibacterial and antifungal cationic peptides have also been used as multilayer components in a number of studies^5,23^. Etienne and coworkers incorporated a defensin peptide produced by *Anopheles gambiae* mosquitoes into a PEM composed of polyglutamic acid (PGA) and poly-L-lysine (PLL)^5^. They found that the growth of *Escherichia coli* cultured on the film’s surface was inhibited by 89% with 10 layers of incorporated defensin peptide compared to the control film. They have also been incorporated into advanced materials such as hydrogels and plasma coatings^24,25^ More recently, a new strategy to obtain antimicrobial wound dressings based on the incorporation of AMPs into PEMs formed of chitosan and sodium alginate onto cotton gauzes was established^9^. The authors studied a variety of peptides and found that each one diffused out of the multilayer at different rates, but that each one enhanced the reduction of *S. aureus* and *K. pneumoniae*, compared to the control film.

Nisin is an AMP produced by Gram-positive lactic acid bacterium, *Lactococcus lactis*. This cationic peptide occurs in five natural variants, the two most common being nisin A and nisin Z which differ by one amino acid in residue, 27-histidine and 27-asparagine, respectively. It also has a low molecular weight of approximately 3.3 kDa^26^. The amino acid sequence of nisin Z is depicted in the Supplementary Material. The accepted antimicrobial mechanism of nisin is based on the depolarisation of charged cell wall components, or hydrophobic interactions, allowing nisin to reach cell membranes where it can bind to lipid II in the cell membrane, resulting in the formation of aqueous transmembrane pores and leakage of intracellular components^27,28^. Nisin can also cause cell wall lysis in certain bacteria, including *Staphylococci*^27^. Nisin is currently approved for use as a safe biological preservative by Food Standards Australia New Zealand (FSANZ), Food and Drug Administration (FDA) and the World Health Organization (WHO) due to its non-toxicity and its bacteriolytic and bactericidal activities^29^. Nisin has also been explored as an antitumor agent^18^.

Nisin is currently used in dairy and meat products, canned foods and beverages^29^. However, like many other antimicrobial compounds, nisin can lose its antimicrobial activity when exposed to adverse physicochemical environmental conditions (i.e. pH and ionic strength or temperature). The antimicrobial activity of nisin can also be reduced in food and emulsion products by its susceptibility to enzymatic degradation as well as via other interactions with food components^29^. As a consequence, strategies to protect and/or delay the release of nisin have been widely explored. Nisin has been investigated for use in packaging materials where it has been mixed with plasticisers and formed into a bulk film^30^. Of further significance is the microencapsulation of these compounds^6,10^ as well as their incorporation into PEMs^31^. In each of these cases, the antimicrobial efficacy of the nisin peptide has been preserved, indicating the usefulness of AMP protection via these encapsulation and incorporation methods.

This work describes the preparation of nisin Z loaded PEMs composed of chitosan (CS) and carrageenan (CAR) prepared via the layer-by-layer (LbL) technique^32^. Chitosan, a cationic polysaccharide, is often used to build multilayered films for pharmaceutical or food based applications because it is biodegradable, non-toxic and biocompatible^33,34^. In addition, it is one of the only known cationic polysaccharides that also possesses mild intrinsic antibacterial properties^34^. It is currently accepted that the antimicrobial activity of chitosan is initiated by the electrostatic interactions between the positively charged amino groups of the chitosan and the negatively charged bacterial membrane. The polyanion component of the multilayer, carrageenan, is a highly sulfated, linear, biodegradable, biocompatible anionic polysaccharide, commercially extracted from red marine algae. Carrageenan biopolymers are used prevalently in the food industry but have also found applications in the area of wound healing and drug delivery systems, specifically for antimicrobial applications^35,36^. This combination of natural polysaccharides aims to protect nisin from the physiological environment to prolong its antimicrobial activity.

The combination of chitosan with a sulphated polysaccharide such as alginate or carrageenan to protect nisin has been utilized in the past, however the biopolymers have primary been used as simple microcapsules^37,38^. Instead, we incorporated Nisin Z into carrageenan/chitosan multilayer films as a film component, with an aim to produce a material that could meet the demand for a natural and biodegradable antimicrobial film for use in the food, pharmaceutical, and biomedical industry. Its incorporation and the formation of the multilayers both with and without nisin Z were investigated using several techniques. These techniques include the in situ technique of attenuated total reflection Fourier transform infrared spectroscopy, ATR FTIR, used previously by our group to characterise PEMs^39,40^. In addition, the thickness of the formed layers was measured and the surface topography and wettability assessed, as these parameters can influence bacterial cell adhesion onto surfaces, and thus can play a role in how the films interact with bacterial cells^41^. The antimicrobial activity of these nisin containing films were also evaluated using two bacterial strains, specifically wild type *Staphylococcus aureus* and a methicillin-resistant variant.

## Results and discussion

The following results and discussion section describe: (i) polyelectrolyte multilayer (PEM) formation using the alternating deposition of up to 9 layers of carrageenan (CAR) and chitosan (CS) including the addition of the net positively charged nisin Z within the film, in place of the fourth chitosan layer; (ii) multilayer characterization of the nisin containing PEMs both with and without a terminating CAR layer (layer 9) and; (iii) the antimicrobial activity of the nisin containing PEMs. The full 9 layer multilayer is referred to as a 4.5 bilayer throughout the text (a CAR/CS pair is a bilayer), where layer 1 is carrageenan, layer 2 is chitosan etc. and where in the case of the nisin containing films, the nisin is counted as layer 8. The pairing of chitosan with sulfated polysaccharides including fucoidan or carrageenan for multilayer formation has been widely studied^42,43^ and the formation conditions used here were chosen to mimic those used previously to successfully form multilayers from fucoidan and chitosan^42,44^.

### Multilayer formation

**Figure 1:**
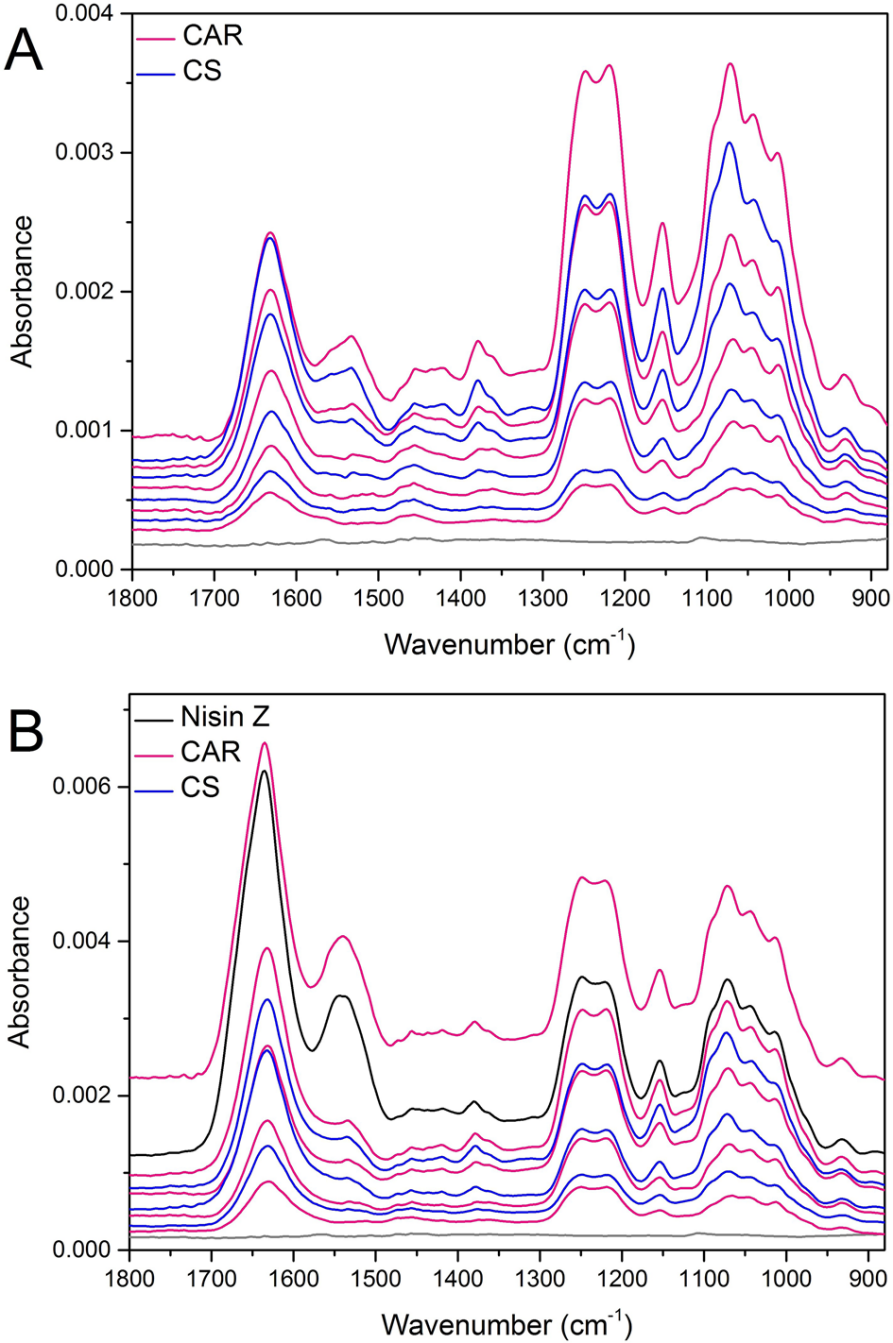
ATR FTIR spectra recorded at each stage of the multilayer build-up for a 4.5 bilayer CAR/CS multilayer (Panel **A**) and for a 4.5 bilayer multilayer that contains nisin Z (Panel **B**). Formation conditions are noted in the text. Blue lines: spectra recorded after CS adsorption. Pink lines: spectra recorded after CAR adsorption. Black line: spectra recorded after nisin Z adsorption.

ATR FTIR spectroscopy was used to obtain quantitative information about multilayer formation, with spectra recorded after each polymer adsorption/rinse step during build-up. ATR FTIR spectra of a 4.5 bilayer CAR/CS film are presented in Fig. 1, panel A. Each spectrum during the build-up has been processed by manually subtracting the spectrum of the background electrolyte to remove the contributions from water in the O–H bending region. The CS layers are represented by blue lines, whereas the pink lines represent the successive CAR adsorption steps. The anchoring PEI layer is represented by the light grey trace. The build-up alternated between the two polysaccharides until the full 4.5-bilayer multilayer was recorded. The ATR FTIR spectra of the 4.5 bilayer CAR/CS film containing nisin with a CAR terminating layer is presented in Fig. 1, panel B*.* The spectra are similar until the fourth polycation layer, which has seen chitosan replaced with the positively charged nisin Z, and is represented by a black trace.

The fingerprint regions within the spectral range of 1800 to 900 cm^−1^ contain the characteristic peaks of interest for the two multilayered systems. Peak assignments are presented in Table 1. The peak assignments were based on solution spectra peak assignments and previous work from our research group^42,44^ as well as from others^45–47^. The majority of the peak assignments are directly comparable to those assigned from the spectrum of the nisin (Fig. S1, Electronic Supplementary Material), and from the CAR and CS solution spectra (Fig. S2, Electronic Supplementary Material). The peaks assigned to acetic acid in the CS solution spectrum are not observed in the multilayer build-up spectra due to its dilution and removal via rinsing during formation.

**Table 1:** Peak positions and assignments for (i) ATR FTIR spectrum of a 4.5-bilayer CAR/CS PEM; and (ii) ATR FTIR spectrum of a 4.5-bilayer CAR/CS PEM containing nisin Z.

Peak Positions (cm^-1^)		Peak Assignment
CAR/CS Multilayer	CAR/CS Multilayer with nisin	
	1634	Amide I^42,45^
1631	1631	ν(C = O), δ(O–H)
	1538	Amide II^42,45^
1453	1453	ϑ(CH_2_)^42^
1421	1421	δ(CH_2_)^42^
1380	1380	δ(CH_3_)^42^
1248	1248	ν_as_(SO_3_^-^)^46^
1219	1219	ν_as_(SO_3_^-^)^46,47^
1154	1154	ν(C–O–C),ν(C-N)^42^
1091	1091	ν_as_(C–O–C)^42^
1072	1072	ν_as_(C–O–C)^42^
1064	1064	ν_as_(C–O–C)^47^
1042	1042	ν_as_(C–O–C)^47^
1014	1014	ν_as_(C–O–C)^47^
931	931	ν(C-O)^47^
839	839	ν(C-O-S)^42^

^a^Peaks dominated by acetic acid used to dissolve chitosan

^b^Most likely from uronic acid present in the carrageenan; Annotations: ν—stretching vibrations, ν_s_—symmetric stretching vibrations, ν_as_—asymmetric stretching vibrations, δ—in-plane bending vibrations, ϑ—bending vibration.

The build-up of both systems with and without embedded nisin proceed as expected, with peaks attributable to CAR, specifically the sulfate peaks at 1248 cm^−1^ and 1219 cm^−1^, increasing at each CAR adsorption step. Likewise, the peaks that are attributable to chitosan also increase at each CS adsorption step; these include those attributable to the glycosidic linkage region between 1154 cm^−1^ and 1012 cm^−1^. Unfortunately, many of these peaks are shared by or in close proximity to those observed for CAR, however the stepwise increase of these peaks for each adsorption step was observed. In the spectra of the multilayer containing nisin Z (Fig. 1, panel B), it was also observed that during the subsequent CAR adsorption step, there is a loss of nisin Z, represented by a decrease in the amide I and amide II bands. This is likely due to stripping of loosely bound peptide by the oppositely charged CAR polysaccharide.

**Figure 2:**
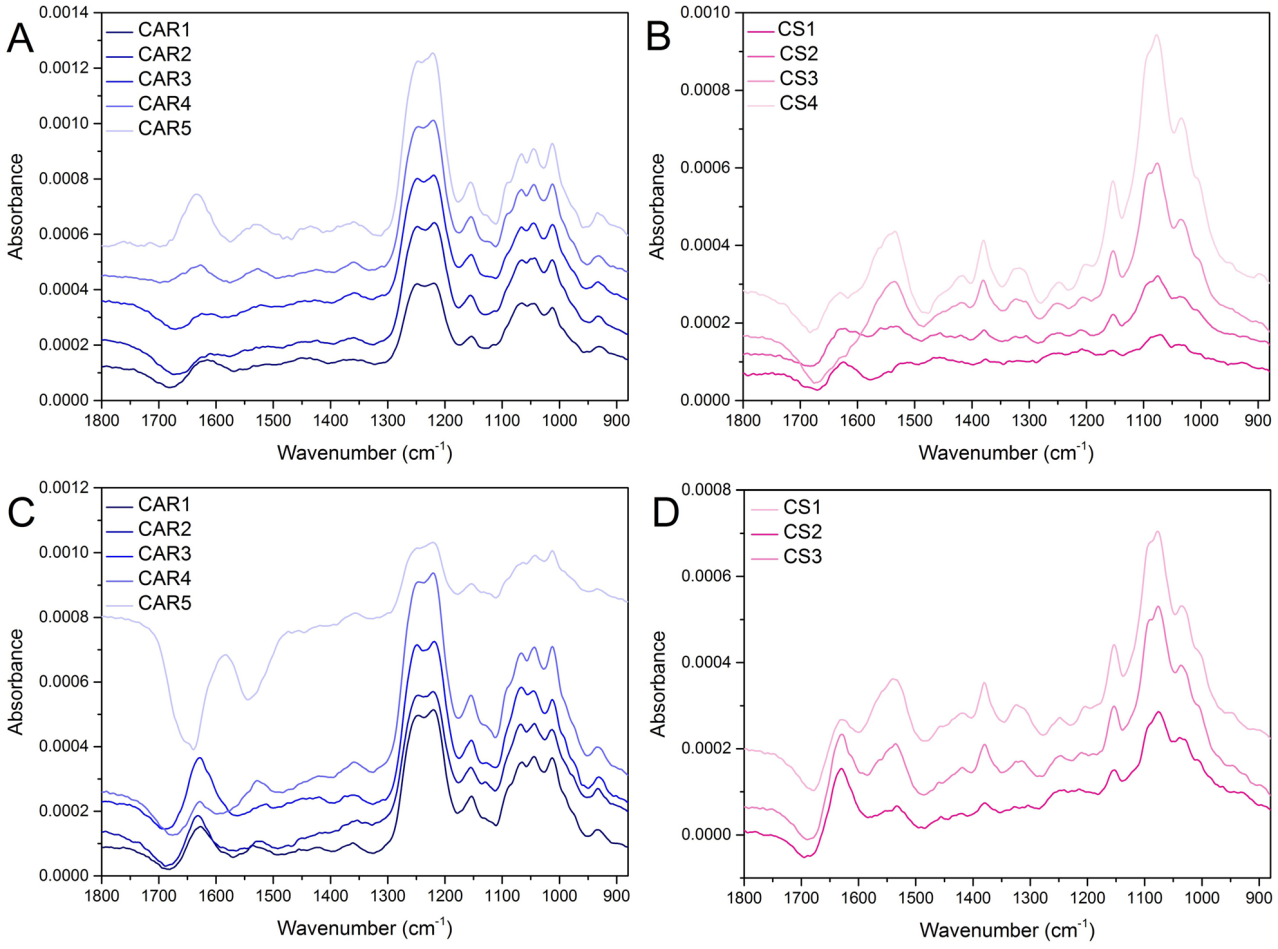
ATR FTIR spectra of discrete polymer layers 1 through 5 and 1 through 6 of the CAR (panel **A**) and of the CS (panel **B**) added to the CAR/CS multilayer without nisin Z and ATR FTIR spectra of discrete polymer layers 1 through 5 and 1 through 6 of the CAR (panel **C**) and of the CS (panel **D**) added to the CAR/CS multilayer that contained nisin Z. Spectra were processed by subtracting successive spectra from the previous spectra in the multilayer stack shown in Fig. 1.

**Figure 3:**
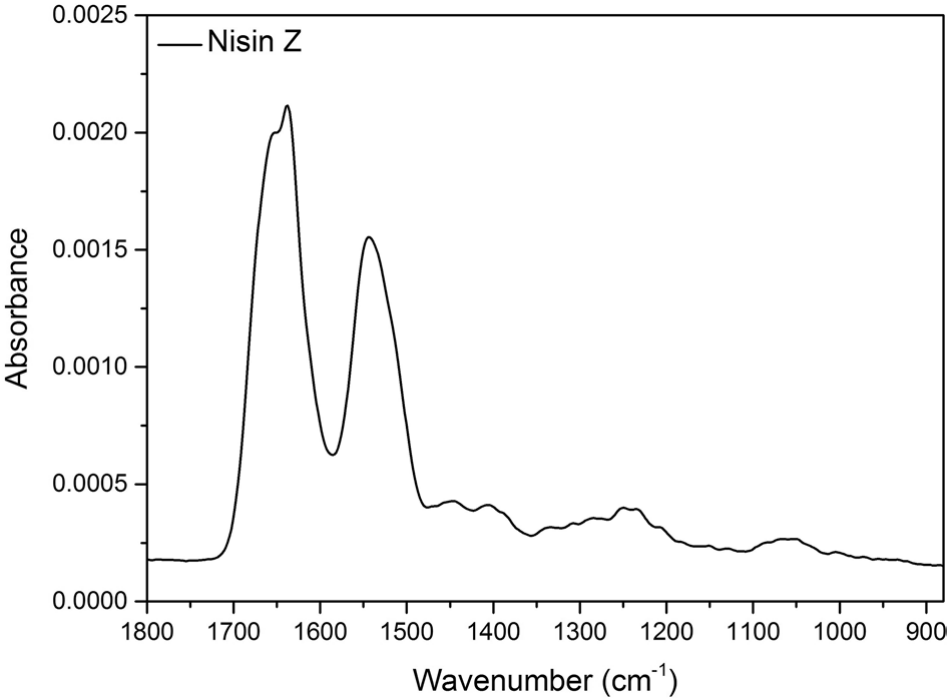
ATR FTIR spectra of discrete nisin Z layer from the CAR/CS multilayer containing nisin Z. The spectrum was processed by subtracting the previous spectrum (CAR4) from the spectrum collected after nisin incorporation in the multilayer stack shown in Fig. 1.

Representative individual layer spectra are presented in panels A and B of Fig. 2 for the CAR/CS system and in panels C and D for the CAR/CS system that contains nisin Z. These spectra are produced by subtraction of the previously acquired spectra at a factor of 1, and show the changes in the multilayer between the acquisition of the two spectra, i.e. a spectrum corresponding to a single adsorbed layer. Additionally, the spectrum collected after the adsorbed nisin Z layer is presented in Fig. 3. Further information can be ascertained from these individual layer spectra, which more clearly display the changes with each subsequent layer in comparison to the multilayer spectra in Fig. 1. It should be noted that the distortions in the O–H bending region of the individual layer spectrum at approximately 1636 cm^−1^ exist due to a decrease of bulk water in the evanescent wave as the film gets thicker, but do not interfere with peaks used for mass quantification. In both multilayer systems, the majority of the peaks present in the CAR and CS individual layer spectra correspond to the peaks assigned to the solution spectra with a few exceptions. The sulfate peaks in the CAR individual layer spectra have undergone some changes when compared to the solution spectrum. A shift of the sulfate peak from 1221 cm^−1^ in the solution spectra to 1219 cm^−1^ in the individual layer spectra can be attributed to ion pairing interactions. In addition, the peak area decreases in relation to the neighbouring sulfate peak at 1248 cm^−1^.

The spectrum of most importance in Fig. 2 is the spectrum of CAR collected after nisin Z addition (see CAR5 in panel C). As noted previously in the full multilayer stack, the negative amide peaks are representative of loosely bound nisin Z that is stripped away by incoming CAR. Also relevant is the deviation of the increasing sulfate band as a function of layer number, suggesting only a small amount of CAR has been adsorbed onto the nisin Z terminated film. The possible reason, while unconfirmed for this system (though observed for defensin^5^) is the low surface charge of the peptide. If this is the case, less CAR is required to reverse the charge of the multilayer film. In addition, while the solution spectrum of CS is dominated by peaks arising from acetic acid, it is possible to see some distortion in the sulfate region of the individual layer spectra in Fig. 2 (panel B and panel D) between 1200 and 1300 cm^−1^. This has been observed by our group in the past for CS in fucoidan/CS multilayers^42,44^ which was attributed to the ion-pairing interactions that altered the sulfate region of the fucoidan layer deposited prior to the CS layer of interest. The same can be said for this system.

Finally, the spectrum of nisin incorporated into the multilayer is displayed in Fig. 3 (obtained by spectral subtraction, similarly to those in Fig. 2). The FTIR data presented here shows that there are some differences between the bulk nisin solution spectrum (Fig. S1, Electronic Supplementary Material) and the spectrum of the nisin Z adsorbed within the multilayer in Fig. 3. Specifically, the amide II band shifts from 1537 to 1544 cm^−1^. One of the more obvious changes is the shift of the amide I band at 1642 cm^−1^ to an average lower wavenumber of 1634 cm^−1^. More specifically, a separation of the amide I band can be seen with two peak maxima at 1652 cm^−1^ and 1638 cm^−1^ which could be due to either (i) changes in the secondary structure of the nisin Z when it is adsorbed *versus* in solution^48^ or (ii) mass loss of either CAR or CS from the underlying layer as nisin adsorbs causing distortion of the peak.

A shift of the amide I band is associated with a change in peptide/protein structure and also hydrogen bonding of certain amino acid residues^45^. The shift of the amide I band has been observed by Wu et al. and was correlated to the electrostatic interaction of nisin Z to polyglutamic acid during the formation of nisin Z loaded nanoparticles^49^. The shift has also been correlated to hydrogen bonding between the N–H of peptides such as nisin Z and the O–H of chitosan^23,50^. The complexation of nisin and chitosan is quite common^6,50^, suggesting that hydrogen bonding likely occurs with CS in the studied system, in addition to the electrostatic interactions between nisin Z and CAR. Given the FTIR data, we hypothesise that the nisin Z, adsorbing onto an oppositely charged polymer and being a relatively small molecule (3.3 kDa), diffuses within the film upon adsorption^51^, a phenomenon that we have observed recently for lysozyme into a similar PEM composed of CS and fucoidan^52^.

Using the required parameters for the calculations (Figure S3 and Table S2, Supplementary Information) as well as the individual layer spectra, the cumulative masses of the two multilayered systems, ${\Gamma }_{FTIR}$ were determined using the method developed by Pitt and Cooper. Also mentioned in the Supplementary Information, the peaks used for the calculations include the sulfate peaks between 1248 and 1219 cm^−1^ for the calculations for CAR as well as the glycosidic linkage peaks between 1160 and 1000 cm^−1^ for the CS calculations. The amide II band at 1538 cm^−1^ was used for the nisin Z adsorbed mass calculations.

**Figure 4:**
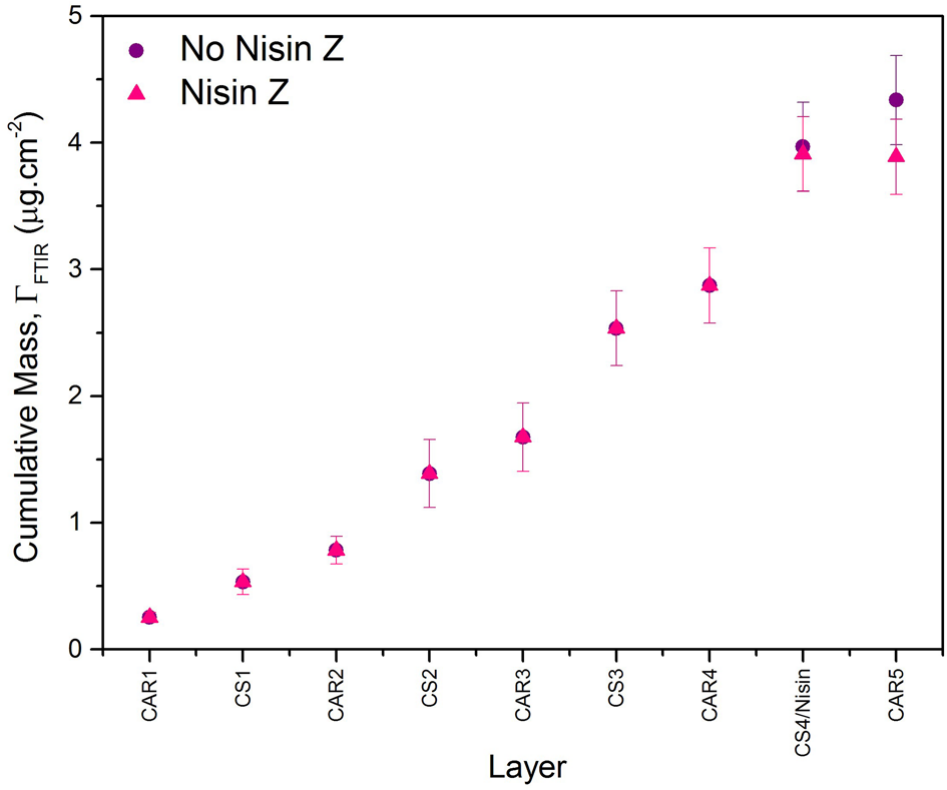
Cumulative mass plots for multilayers of CAR/CS (purple circles) and CAR/CS containing nisin (pink triangles) formed on a solid support determined using ATR FTIR. Cumulative masses were determined from three independent experiments.

The cumulative mass of the two multilayered systems terminated with CAR is presented in Fig. 4. The 4.5 bilayer CAR/CS system represented with the purple circles was calculated to have a total mass of 4.34 ± 0.35 µg cm^−2^, and the CAR/CS system containing nisin Z with a terminating layer of CAR, represented by the pink triangles was calculated to have a total mass of 3.89 ± 0.30 µg cm^−2^. Also of interest in this work is the nisin Z containing system without the terminating CAR layer, calculated to have a total mass of 3.91 ± 0.30 µg cm^−2^$.$ Accounting for the nisin lost during the CAR adsorption step (0.147 ± 0.043 µg cm^−2^), the mass of nisin contained within the film was determined to be 0.89 ± 0.064 µg cm^−2^. Thus, the nisin Z makes up approximately 22.8% of the film’s total mass. Both films (with and without nisin Z) display supra-linear growth. This type of growth is commonly observed for natural polysaccharide polymers, which are more likely to experience greater polydispersity, as well as diffusion of adsorbing species into the bulk of the film^39,40^.

Marvdashti et al.^30^ explored the activity of a bioactive polyvinyl alcohol-*Alyssum homolocarpum *seed gum**-**nisin composite film. Films containing 120 µg cm^−2^ nisin were able to reduce approximately 90% of the microbial population of *S. aureus* after 6 days of storage, whilst films with 400 µg cm^−2^ nisin, were able to reduce 100% of the microbial population of *L. monocytogenes* and *S. aureus* after as little as 48 h and up to 6 days of storage. This is significantly (~ 130–450 times) larger than the amount incorporated into the CAR/CS multilayer in our study. While it should be noted that film properties and the interaction of nisin Z with other film components will dictate its antimicrobial activity, given the results in the study mentioned above, it is possible that such a small adsorbed amount may not have a substantial effect on microbial growth, and this was our motivation to undertake antimicrobial testing of the nisin Z incorporated film.

### Multilayer characterization

Considering that surface properties can influence bacterial attachment, the surface properties of the multilayers were investigated prior to undertaking studies of antimicrobial efficacy. These multilayers include the 4 bilayer CAR/CS film with a nisin Z outer layer (sample A), the 4.5 bilayer film with nisin Z/CAR outer layer (sample B) as well as the 4.5 bilayer CAR/CS film containing no nisin Z (sample C). The wettability of fully hydrated samples was determined by means of receding water contact angles (Fig. S4, Electronic Supplementary Material). All three samples were found to be hydrophilic, with the receding water contact angle, ${\theta }_{rec}$, between $22^\circ \pm 2^\circ$ and $25^\circ \pm 2^\circ$. Hence the wettability is similar for all three sample architectures. It is therefore unlikely the wettability would be the driving factor differentiating bacterial attachment for the studied multilayer architectures.

The thickness of each PEM sample was determined using AFM to image across a scratched section of the sample (Fig. S5, Electronic Supplementary Material). The samples all remain relatively similar, where samples A, B and C have increasing thicknesses of 21.8 nm, 24.9 nm and 26.1 nm, respectively (Table 2). Sample A has the smallest thickness as expected. Samples B and C, with an additional CAR layer are both slightly thicker than A. Sample C, containing no nisin Z, is thicker than both samples A and B. Relating these thickness values with FTIR information, for the nisin Z containing films, upon the addition of CAR, loosely bound nisin Z is stripped away and only a very small amount of CAR is adsorbed on to the film. In comparison, sample C was able to continue its growth uninterrupted.

**Table 2:** Multilayer thickness and surface roughness parameters for CAR/CS films with (sample A and B) and without nisin Z (sample C).

Sample	A	B	C
PTV (nm)	185.4	194.2	229.2
R_RMS_ (nm)	15.9	16.3	22.3
R_average_ (nm)	8.9 ± 0.2	9.4 ± 0.2	13.3 ± 0.4
Ironed surface area (μm^2^)	4.90 ± 1%	5.17 ± 1%	5.46 ± 2%
Thickness (nm)	21.8 ± 0.5	24.9 ± 0.5	26.1 ± 0.7

**Figure 5:**
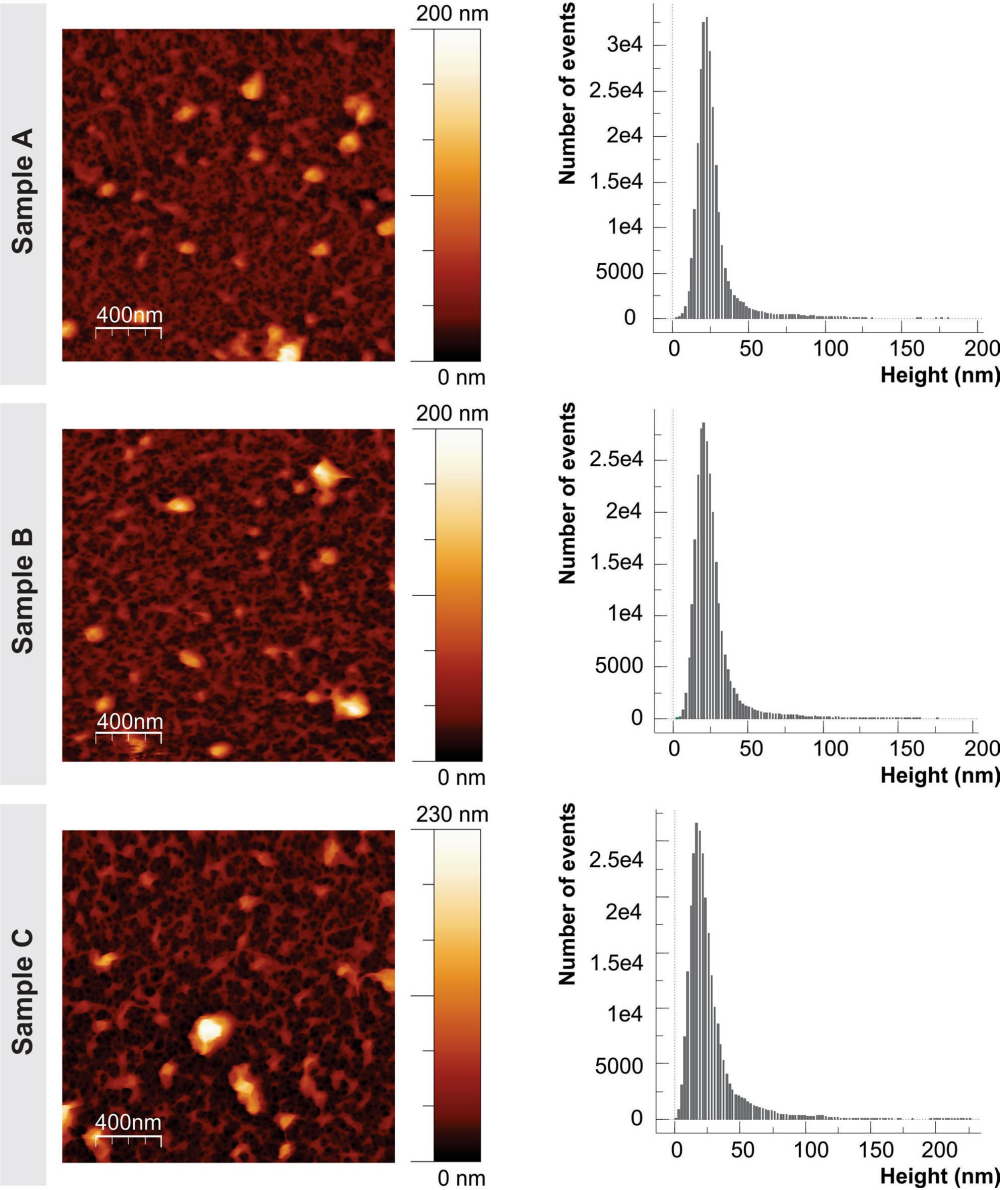
AFM 2 × 2 μm^2^ topography images collected in 0.1 M KCl solution are shown on the left. The height histograms of relevant samples are shown on the right. Sample (**A**): PEM with nisin Z outer layer; Sample (**B**): PEM with nisin Z/CAR outer layer and; Sample (**C**): PEM without nisin Z.

The representative 2 × 2 µm^2^ images of surface topography of samples A, B and C collected in 0.1 M KCl can be seen in Fig. 5. The height histograms are similar for all three samples, however there are some differences in roughness parameters (Table 2), with sample A being characterized by the lowest root-mean-square (R_RMS_) and average (R_average_) roughness values (15.9 nm and 8.9 nm, respectively). In addition, the difference between the surface area of a smooth 2 × 2 µm^2^ and ‘ironed’ 2 × 2 µm^2^ surface area of sample A is the smallest among the three studied samples, with the ‘ironed’ 2 × 2 µm^2^ surface area of sample A being equal to 122.5% of that of a smooth surface of the same size. Sample B has the values of R_RMS_ and R_average_ (16.3 nm and 9.4 nm, respectively) comparable to those for sample A, however there is noticeable increase in the ‘ironed’ surface area (129.3% of that of a smooth surface of the same size and 8.8% larger than for sample A). Sample C is the roughest, with R_RMS_ and R_average_ being 22.3 nm and 13.3 nm, respectively. The ironed surface area is the largest too—136.5% of that of a smooth surface of the same size. Surface roughness increases from sample A to sample C. The differences are relatively small and they do not suggest that surface topography (or roughness to be precise) would have a significant impact on bacterial adhesion for these three different multilayer architectures.

Interestingly, during sample preparation for the AFM experiments, we have observed gas nucleation (the formation of gas bubbles from dissolved gas molecules, associating at or adjacent to hydrophobic material) on samples A and B (but not on sample C, i.e. the sample without embedded nisin Z—see Fig. S6, Electronic Supplementary Material) when they were transferred from the fridge (4 °C) to room temperature (22 °C). Since the nucleation did not occur on all three samples, this effect cannot be attributed to the decrease of gas solubility at higher temperature.

Yang et al*.*^53^ describes the parameters facilitating small bubble formation at solid/water interfaces. Among them are surface heterogeneity (i.e. local differences in surface free energy) and wettability. It has been known for decades that increasing surface roughness^54^ and hydrophobicity^55^ of the samples can affect gas nucleation^56^. Bubble nucleation from ‘gas cavities’ is also a common concept^57^. Gas cavities refer to grooves or defects at the surfaces where the formation of bubbles at these sites is dependent on a number of factors including the nucleation spot size, shape and wettability. Given that the surface roughness is similar (albeit, we note that the sample of the highest roughness is the sample C; surprisingly, this is the only sample on which the gas nucleation is not observed) for all three sample architectures and that all samples are hydrophilic, as manifested by low values of the receding water contact angle, the presence of the gas bubbles must be attributed to the chemical differences with the film, i.e. the presence of nisin Z.

In relation to the studied multilayers, on a molecular level, a number of ‘defects’ likely exist between the loops and chains of the polysaccharides. The inclusion of the nisin Z within the films may impart regions of hydrophobicity, where certain regions of the nisin peptide (specifically across the *N*-terminal) contain hydrophobic residues^58^. Although the receding water contact angle measurements showed no differences in macroscopic surface hydrophobicity, the scale at which these hydrophobic regions exist within the multilayer may not have affected the wettability of the uppermost surface. Thus, the nucleation of gas observed for samples A and B, likely originate from the hydrophobic domains of the nisin Z peptide within these multilayers, where the gas ‘nuclei’ can be formed. Such gas ‘nuclei’ can then grow to form a sub-millimetre bubble at the multilayer/solution surface.

### Antimicrobial activity

**Figure 6:**
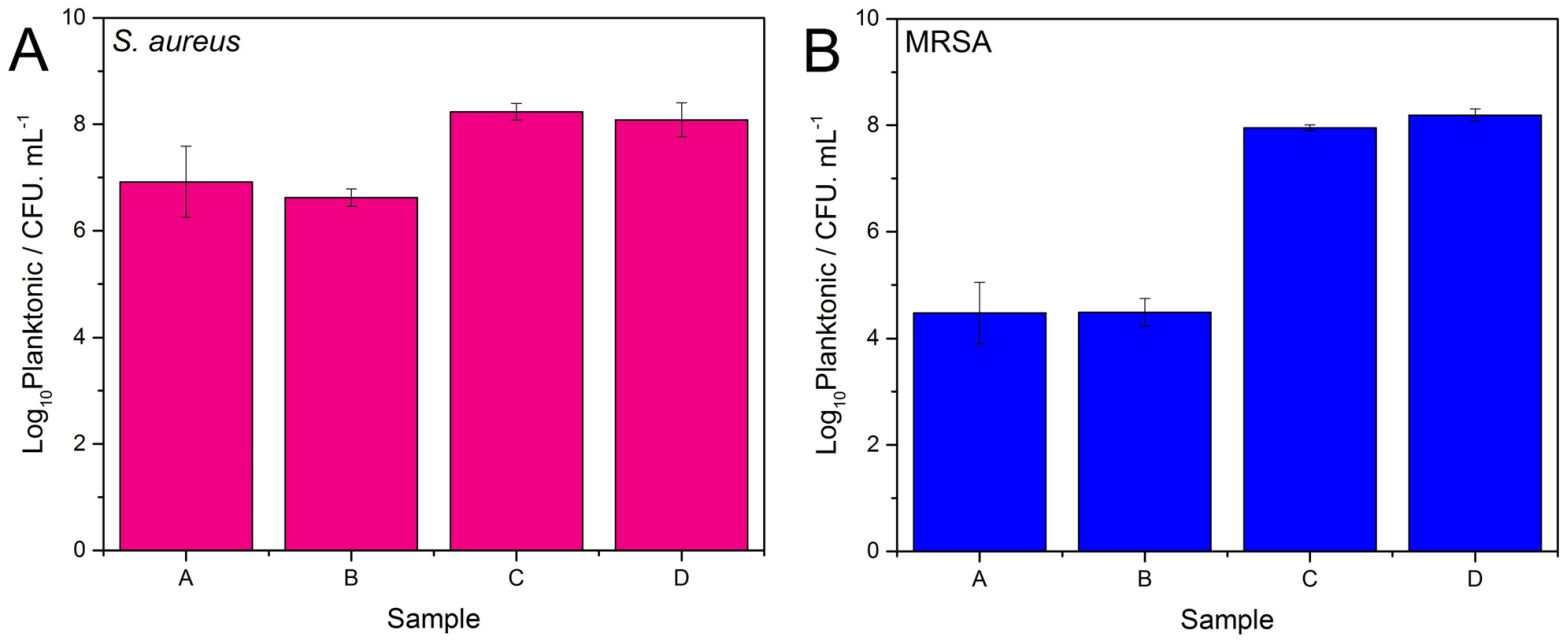
Viability of S. aureus (Panel **A**) and MRSA (Panel **B**) planktonic cells in the presence of the glass slides. Sample (**A**): PEM with nisin outer layer; Sample (**B**): PEM with nisin/CAR outer layer; Sample (**C**): PEM without nisin and; Sample (**D**): Blank glass slide. The data are from a minimum of two biology experiments, with two replicates in each (i.e. at least 4 measurements in each case).

**Figure 7:**
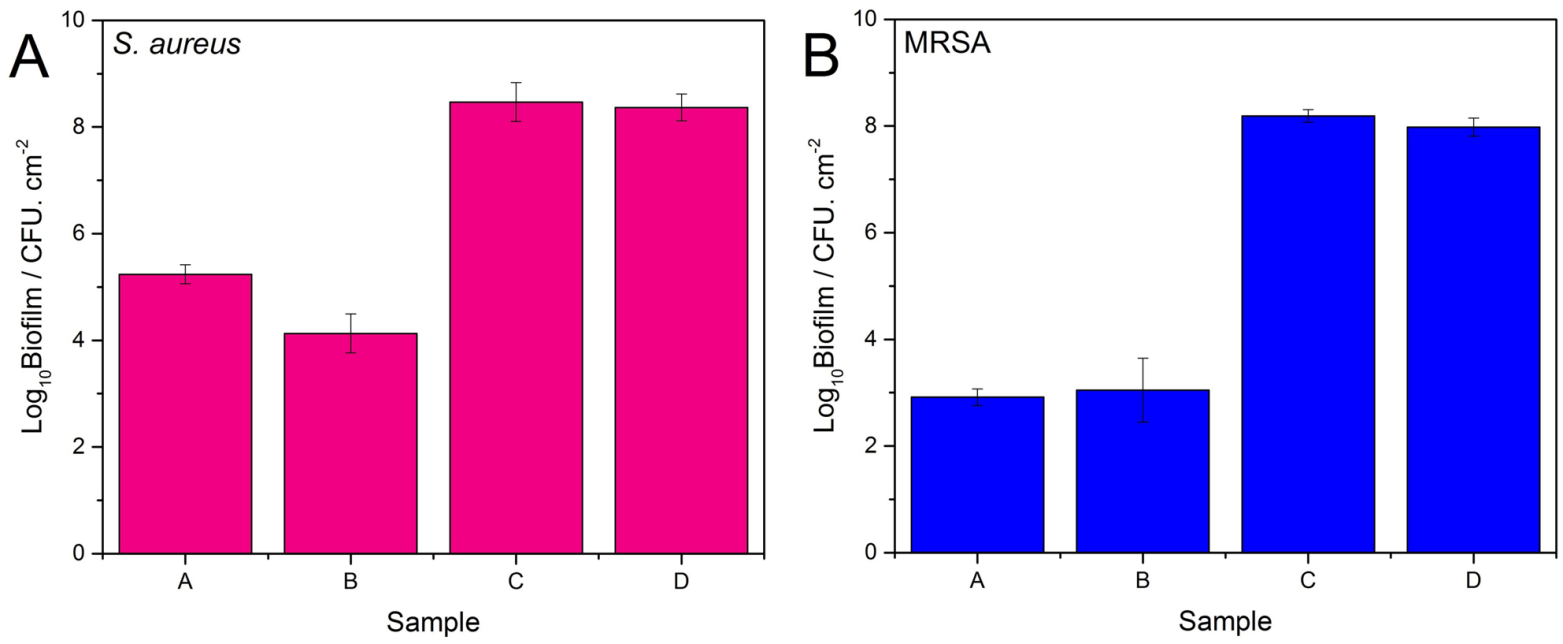
Viability of S. aureus (Panel** A**) and MRSA (Panel** B**) biofilm cells in the presence of glass slides. Sample (**A**): PEM with nisin Z outer layer; Sample (**B**): PEM with nisin Z/CAR outer layer; Sample (**C**): PEM without nisin Z and; Sample (**D**): Blank glass slide. The data are a minimum of two biological replicates. Note: the biofilm cells were formed in situ from the planktonic cells during the course of the 6.5 h incubation. The data are from a minimum of two biology experiments, with two replicates in each (i.e. at least 4 measurements in each case).

The bactericidal activities of the studied nisin Z containing PEMs against two bacterial strains—*S. aureus* and MRSA—were determined using CFU analysis under static conditions. The viability of planktonic and biofilm *S. aureus* and MRSA bacteria after 6.5 h of incubation in the presence of samples A, containing a 4 bilayer CAR/CS film with a nisin Z outer layer and B, containing a 4.5 bilayer film with nisin Z/CAR outer layer are compared in Fig. 6 and Fig. 7. The initial bacteria loading was 10^5^ CFU mL^−1^. Two control substrates, sample C which contains 4.5 bilayer CAR/CS PEMs without nisin Z and sample D which is the uncoated glass substrate, were included for comparison.

Figure 6 displays the planktonic viability data for all four samples for *S. aureus* (Panel A) and MRSA (Panel B). The CFU data is tabulated in Table S3, SI. We observed more than 1-log_10_ and 3-log_10_ reductions in CFU (which equates to 93.16% and 99.97% reduction in number of viable cells) of *S. aureus* and MRSA, respectively, for nisin Z containing PEMs samples A and B compared to the control substrates C and D. This result is interesting because as discussed earlier, we expect the nisin Z to be bound via electrostatic and hydrogen bonding interactions onto and into the PEM. Thus, no nisin Z leaching is expected from the film, contradicting this planktonic viability data. However, it is likely that the incubation of the multilayers into the bacteria solution which is composed of cell culture media is leading to film degradation (mass loss) and possible nisin Z loss from the film. While this has not been confirmed for this system, the same phenomenon has been seen by our group for films composed of chitosan and fucoidan^52^ upon their immersion in PBS solution, where pH (and ionic strength changes) caused both swelling and mass loss from the films.

Figure 7 displays the *S. aureus* (Panel A) and MRSA (Panel B) biofilm data for the studied samples. The CFU data is tabulated in Table S4, SI. The *S. aureus* biofilm data in Panel A displays 3.2 and 4.3-log_10_ reductions in CFU (which corresponds to 99.93 $\%$ and 99.99 $\%$ reductions in viable cells) for samples A and B respectively compared to the control samples. In addition, the MRSA biofilm data in Panel B displays high biofilm reductions of 99.99 $\%$ for both samples A and B.

Based on AFM and wettability data, it can be concluded that the biofilm attachment differences between samples is not related to the surface properties of the PEMs, because the surface topography (the surface roughness) and wettability are very similar for all three sample types. Hence the antimicrobial effect manifesting in the different samples must be solely due to the difference in chemistry of the multilayers, confirming the efficiency of nisin to prevent bacterial attachment regardless of whether it is the terminating layer or whether it is protected by an outer layer of CAR.

In addition, the lower viable biofilm cells seen for samples A and B is not entirely unexpected, as the antimicrobial activity of nisin Z against Gram-positive bacteria such as *S. aureus* has been reported in the literature for various nisin containing systems^30,38,59^. It should be noted that sample C, the control CAR/CS film, displayed no bactericidal activity. While chitosan is said to possess intrinsic antibacterial properties^29,42^ and has been shown to display moderate antimicrobial activity against *S. aureus* in the past^60^, it is possible that it was not available to interact with bacteria in the same way nisin Z was. It is likely that the charges that are required to interact and penetrate the bacterial cell wall were not freely able to do so. The lack of activity for sample C also makes clear that the reduction seen for samples A and B must originate solely from nisin Z. Thus, the CFU results suggest two possible scenarios where the nisin coatings could either prevent the formation of biofilms (i.e. no biofilm attachment) or the nisin coatings are killing the formed biofilms on the surface (hence biofilm survival). Either scenario could yield the observed CFU data displayed in Fig. 6 and Fig. 7. To determine which scenario is true, biofilm imaging experiments were performed, to visually observe biofilm density. In correlation with the CFU data, if the first scenario was to occur minimal formation of biofilms would be expected. On the other hand for the second scenario, one would still observe biofilms on the surface, albeit the majority of the cells in the biofilm would be dead cells.,.

The antibiofilm activity of the two nisin Z containing PEMs against *S. aureus* and MRSA was analysed using a tomographic light microscope (Nanolive 3D Cell Explorer) with the collected images displayed in Fig. S7, Electronic Supplementary Material. After 6.5 h of incubation time in the bacterial culture, light microscopy images of the control samples (panels C and D) display extensive biofilm attachment and colonisation compared to those samples containing nisin Z (panels A and B).

**Figure 8:**
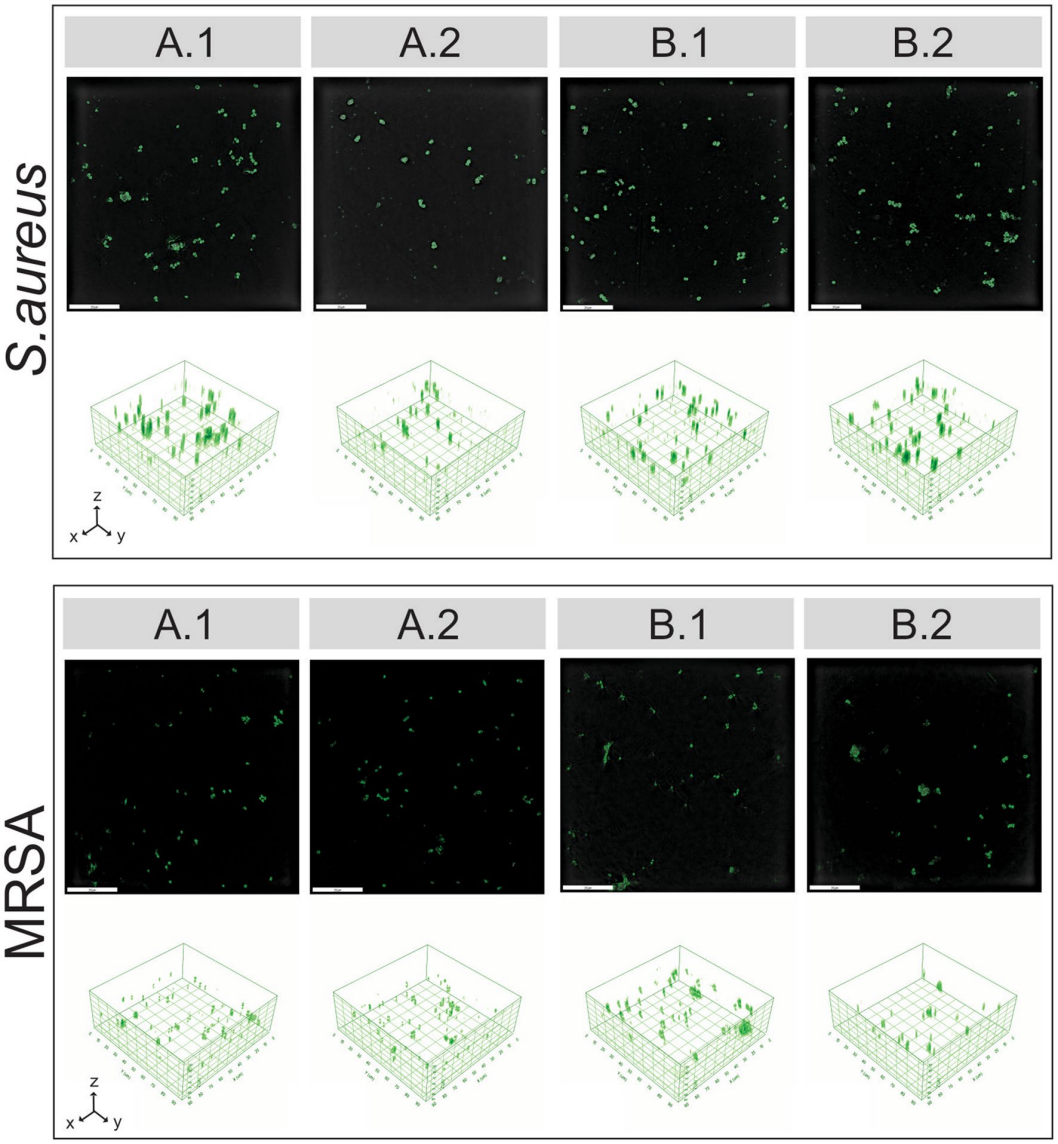
Two-dimensional and three-dimensional topographic microscopy images of S. aureus (top) and MRSA (bottom) attachment onto the multilayered samples containing nisin are displayed in the top and bottom rows, respectively. A: PEM with nisin outer layer and; B: PEM with nisin/CAR outer layer. Numbers represent different samples of the same type. Scale bar = 20 µm. Note: green indicates both live and dead bacteria.

The two- and three-dimensional tomographic microscopy images of *S. aureus* and MRSA cell attachment onto the multilayered samples containing nisin Z are displayed in Fig. 8. The images for both bacterial strains further confirm that the nisin containing samples (A and B) indeed have lower biofilm density than the control samples (C and D), observed using the tomographic light microscope. This most likely implies that the nisin containing films exert their antibiofilm activity via inhibition of biofilm formation. This is not entirely unexpected, where inhibition of biofilm growth with nisin and nisin derivatives has been observed in the past for a variety of gram positive bacterial strains^59,61^.

## Conclusion

The aim of this study was to investigate the incorporation and antimicrobial properties of nisin Z into a natural polyelectrolyte multilayer. The data presented here shows that up to 0.89 µg cm^−2^ of nisin was retained within the CAR/CS film. Carrageenan and chitosan have also proven to be appropriate choices for antimicrobial thin film applications, whereby the inclusion of nisin into the CAR/CS multilayer has proven an effective method to protect the AMP, while maintaining its inherent antimicrobial properties. While the mechanism of the antimicrobial activity remains uncertain, (i.e. whether the film is contact-killing or whether the nisin is released within the solution), the nisin containing multilayers retained their antimicrobial activity against both *S. aureus* and MRSA over 6.5 h. Multilayers containing nisin were able to reduce viable planktonic cells for both studied bacterial strains by over as well as inhibit biofilm formation on the multilayers. The work undertaken here is a step towards realising the antimicrobial potential of CAR/CS multilayers for applications within a number of industries including for food and biomedical/pharmaceutical applications.

## Materials and methods

### Materials

Polyethyleneimine (PEI; Branched, 25 kDa), carrageenan (CAR; mix of κ-, and λ-carrageenan, high molecular weight) and chitosan (CS; high molecular weight, > 75 $\%$ deacetylated, 800–2000 cP, 310–375 kDa) were purchased from Sigma Aldrich (Australia). Nisin Z P (ultrapure nisin Z) (≥ 95 $\%$ purity, with the majority of the remaining 5% being moisture, 3.3 kDa) was purchased from Handary SA (Belgium).

Glacial acetic acid (97 $\%$ AR grade), ethanol (100% undenatured, 99.5% v/v, AR grade), sulfuric acid (H_2_SO_4_, 98% w/w, AR grade), 30 $\%$ hydrogen peroxide (H_2_O_2_, AR grade) and potassium chloride (KCl, 99% AR grade) were supplied by Chem-Supply (Australia). Purification of KCl was performed by calcination at 550 °C for 8 h, followed by re-crystallization and a second calcination before its use, to remove any organic impurities. 0.1 M potassium hydroxide (KOH, volumetric grade) and hydrochloric acid (HCl, volumetric grade) were purchased from Sharlau (Spain) and used for the pH adjustment.

*Staphylococcus aureus* ATCC 29213 strain was supplied by In Vitro Technologies, while the *Staphylococcus aureus* 60 (MRSA) was received from the group of Mark Willcox^62^. Other materials used for antimicrobial analysis include tryptone soya broth, which was supplied by Thermo Fisher Scientific (Australia), phosphate buffered saline (PBS), obtained from Life Technologies (Australia) as well as the Luria Bertani (LB) agar, which was sourced as a powder from Sigma Aldrich (Australia).

### Solution and substrate preparation

All aqueous solutions were prepared with Milli-Q water (Advantage A10 system, Millipore, USA) with resistivity of 18.2 MΩ cm, an interfacial tension of 72.4 mN m^−1^ at 22 °C, and a total organic carbon component of no more than 4 ppb. Background electrolyte solution used in all experiments was 0.1 M KCl, and was pH adjusted to pH 6before use. 500 ppm PEI solutions were prepared in 0.1 M KCl for use as an initial layer in film formation. PEI was used at its native pH within 5 days of its preparation. CS was first dissolved in 1% acetic acid at a concentration of 10,000 ppm and stirred overnight. This solution was diluted to 100 ppm in 0.1 M KCl solution before its use. 100 ppm CAR solutions were prepared in 0.1 M KCl and stirred overnight. 2500 ppm nisin Z solutions were prepared immediately before the experiment and stirred briefly to dissolve the polypeptide. CS, CAR and nisin Z solutions were each pH adjusted to pH 6 before their use, and were all used within 24 h of preparation.

A zinc selenide (ZnSe) total internal reflection element, IRE (Harrick Scientific, USA) was used for the ATR FTIR experiments. The ZnSe IRE was polished using OP-U colloidal silica suspension on a MD-Napt 250 mm polishing pad (both Struers, Denmark) in a figure-of-8 motion for 2 min followed by polishing in Milli-Q water on the pad for a further 2 min. The IRE was then sonicated for 15 min in 2 $\%$ pH 7 Tickopur R 30 surfactant (Bandelin, Germany), rinsed with Milli-Q water and then sonicated for another 15 min in ethanol. It was then rinsed thoroughly with Milli-Q water and dried under a stream of N_2_ (99.999%, BOC, Australia) prior to its use.

1 × 1 cm^2^ glass substrates were cut from microscope slides (Paul Marienfeld GmBH, Germany)0.1.5 × 1.5 cm^2^ were cut from silicon wafer (p-type, < 100 > , Si-Mat Silicon Materials, Germany) (approximately 1.5 × 1.5 cm^2^). These two types of substrates were used for the antimicrobial studies and for AFM and contact angle measurements, respectively. The glass substrates were cleaned via immersion in 1 M KOH for 30 min, washed with Milli-Q water to remove any residual KOH from the surface, dried with N_2_ gas and then cleaned in air plasma (Harrick Scientific) for 2 min. Silicon wafers were cleaned via immersion in a piranha (1:3 v/v H_2_O_2_:H_2_SO_4_) solution for 30 min, before being rinsed with copious amounts of Milli-Q water, dried with N_2_ and then air plasma cleaned for 1 min.

### Multilayer formation

There are three distinct multilayer architectures formed within this study. The first was the multilayer system used as a control, containing no nisin Z: i) PEI/(CAR/CS)_4_/CAR. The two others had nisin Z incorporated into them. They are denoted as: ii) PEI/(CAR/CS)_3_/CAR/nisin, and iii) PEI/(CAR/CS)_3_/CAR/nisin/CAR. For the sake of consistency, where necessary the multilayers are labelled alphabetically: A) PEM with a nisin Z outer layer (architecture ii); B) PEM with a nisin Z/CAR outer layer (architecture iii); C) PEM without incorporated nisin Z (architecture i). For the antimicrobial tests, a fourth type of sample was also utilized as a control—a bank glass or silicon wafer surface with no PEM (denoted as sample ‘D’).

For the in situ ATR FTIR experiments, multilayers were formed in the following manner: (i) an initial adsorption of PEI for 15 min; (ii) 5 min rinse in 0.1 M KCl pH 6 (rinsing step); (iii) adsorption of the polycation, CS for 15 min; (iv) 5 min rinse in 0.1 M KCl pH 6; (v) adsorption of the polyanion, CAR in 0.1 M KCl pH 6 for 15 min; followed by (vi) a second 5 min rinsing step. These four steps (iii-vi) were repeated until the desired bilayer number was achieved, with incorporation of nisin Z occurring in place of step (iii) CS when desired.

### Attenuated total reflection (ATR) FTIR

ATR FTIR experiments were performed using a Varian 670 IR spectrometer. The spectrometer had mounted within it a sampling accessory for attenuated total reflection measurements (Fast-IR, Harrick), with a zinc selenide reflection element. The accessory was also equipped with a flow cell, which was used to deliver solution to the crystal surface for adsorption studies. Liquid handling was managed with a peristaltic pump (Masterflex L/S, Masterflex Tygon tubing). The flow rate for adsorption was selected to be 0.333 mL∙min^-1^. This flow rate was increased by a factor of three for rinsing stages of the multilayer build-up. Spectra were acquired with 256 scans of the spectrometer interferometer, and the resolution chosen for the acquisition was 4 cm^−1^. The following order of spectra acquisition was undertaken for the sample: (i) background spectrum of a clean ZnSe crystal in air; (ii) spectrum of water vapour (recorded 30 min after the background spectrum – acquired for data post-processing); (iii) spectrum of KCl electrolyte solution (for post spectral processing); (iv) the same sequence for solution flowing was used as described previously, where spectra were recorded after each rinsing step. For the preparation of the calibration curves, spectra were recorded for the bulk solution as well as after each successive KCl rinse, in order to be used to account for any polymer that had adsorbed onto the ZnSe. The OMNIC spectral processing software package (Thermo Fisher Scientific, Australia) was used to analyse and process the acquired spectra where the spectra were processed post collection to remove contributions from the background electrolyte and water vapour, where required. Throughout the text, representative data sets are displayed, though each ATR FTIR experiment was performed as three independent repeats.

### ATR FTIR spectroscopy for mass quantification

Quantification of adsorbed polymer and macromolecule mass was attained through the method proposed by Pitt and Cooper^63^, a method previously used by our group^44^. The method does not rely on layer refractive index nor layer density and is reliant only on peak absorbance and the attainment of a concentration calibration curve. Thus, using the spectra acquired throughout multilayer build-up in combination with a calibration curve, one is able to quantify the mass of each individual adsorbed polymer as well as the total mass of the PEM itself.

### Atomic force microscopy

AFM is a powerful technique providing information about the structure of adsorbed polymer in sub-monolayers^64,65^ as well as topography of polymer films^66^ in a liquid environment. PEM thickness and topography were determined using a Bruker Multimode 8 AFM with a Nanoscope V controller (Bruker, USA) which was located on an anti-vibration table (Vision IsoStation, Newport, USA). The AFM was used with a vertical engagement ‘J’ scanner with a maximum *x*–*y* translation of 125 µm and a *z*-range of 20 µm. The images were collected in PeakForce Tapping mode 0.1 M KCl at ambient temperature (~ 22 °C) using a standard quartz liquid cell and silicon nitride cantilever of a nominal spring constant of 0.7 N m^−1^, a nominal tip radius of 2 nm, and resonance frequency of 150 kHz (SCANASYST-FLUID + , Bruker, USA).

Thickness measurements were achieved by scratching the PEM with the tip of a stainless steel needle in order to expose the silicon wafer substrate beneath (previous work by our group has shown no damage to the underlying silicon/native silica layer using this methodology^52^). The edge of a scratch was identified with an optical microscope and scanned to reveal film thickness (i.e. the height difference between the exposed silicon wafer substrate and the PEM) as well as the surface topography of the multilayer. The scan size for each image was set either to 5 × 5 μm^2^ (for film thickness determination) or 2 × 2 μm^2^ (for topography measurements). All collected images were analysed using WSxM 5.0 Develop 9.1 software (Madrid, Spain)^67^, while the thickness was extracted using SPIP 6.6.1 (Hørsholm, Denmark). Images were acquired for three multilayer architectures, for two independent repeats for at least three positions per independent sample for both thickness and topography measurements.

### Contact angle measurements

Contact angle measurements, specifically the static receding water contact angles, ${\theta }_{rec}$, were determined using the captive bubble geometry with an OAH 200 instrument (DataPhysics, Germany) located on an anti-vibration table (Vision IsoStation, Newport, USA). Measurements were achieved by depositing a small (3–5 μL) air bubble on the surface of each sample using a gastight micro-syringe (Hamilton, USA) fitted with a u-shaped stainless steel needle that released the bubbles vertically upwards through the solution to meet the upturned samples. The bubble shape was then fitted with the Young–Laplace equation to extract the receding water contact angles.

### Antimicrobial study

Bactericidal activity of the multilayers was determined against two Gram-positive bacterial strains, *S. aureus* ATCC 29213 (In Vitro Technologies) or *S. aureus* 60 (MRSA)^62^*.* A single colony of either *S. aureus* ATCC 29213 or *S. aureus* 60 was cultured at 37 °C overnight. The medium used was tryptone soya broth (10 ml, TSB, Thermo Fisher Scientific, Australia). Once cultured, the colonies underwent a 1:1000 dilution with additional tryptone soya broth, prior to the dispensing of the suspension as 1 mL aliquots in a multi-well plate. In each plate was also placed a single glass substrate coated with the multilayer material. Incubation of the plates was undertaken under static conditions for six and half hours at 37 °C. Planktonic and biofilm bacteria viability was ascertained via the drop plate method. For planktonic analysis, free-floating cells in the biofilm supernatant were collected, serially diluted in sterile phosphate-buffered saline, PBS (Life Technologies Australia) and plated onto Luria–Bertani (LB) agar (powder, Sigma Aldrich). The procedure for biofilm analysis was different. Loosely adhered bacteria were removed from the surface of the samples by washing with sterile PBS (twice). The samples were then placed in clean wells in a new well plate, after which the biofilm was harvested from the substrates by immersing them in sterile PBS (1 mL), subjecting them to ultrasonication for 20 min (150 W, 40 kHz; Unisonics, Australia). The released/suspended biofilm material underwent serial dilution in sterile PBS, and then plated onto LB agar. Biofilm colonies were counted, and the biofilm colony-forming unit, CFU, was calculated after overnight incubation at 37 °C. All experiments were repeated in at least two independent experiments.

### Biofilm imaging

In a similar manner as the killing study, either *S. aureus* ATCC 29213 or *S. aureus* 60 biofilms were grown in the presence of different PEM systems. After 6.5 h incubation, the surface of the glass substrate (surface area, 1 cm^2^) was washed with sterile PBS to remove loosely attached bacteria. The coverslips were then mounted on 35 mm tissue culture dishes (FluoroDish, World Precision Instruments Inc., Sarasota, FL, U.S.A.) using PBS as the media and imaged with a tomographic microscope (3D Cell Explorer, NanoLive, Lausanne, Switzerland) equipped with digital staining software (as part of the instrument control software) to determine biofilm attachment on the coverslips. Images were taken with the best focus from different locations, and all experiments were repeated in at least two independent experiments.

## Supplementary Information

Supplementary Information.

## Data Availability

The datasets generated during and/or analysed during the current study are available from the corresponding author on reasonable request.
