# Supplementary material for: Incorporation and antimicrobial activity of nisin Z within carrageenan/chitosan multilayers
Source: Sci Rep. 2021 Jan 18;11:1690. doi: 10.1038/s41598-020-79702-3 (PMC7814039; doi:10.1038/s41598-020-79702-3)
Supplement: Supplementary file 1 — Supplementary Information. [file 41598_2020_79702_MOESM1_ESM.docx]

# Supplementary Information:

# Incorporation and antimicrobial activity of nisin Z within carrageenan/chitosan multilayers

Jessie L. Webber^†1,2^, Rashin Namivandi-Zangeneh^†3^, Slawomir J. Drozdek^1,4^, Kazimiera A. Wilk^4^, Cyrille Boyer^3^, Edgar H. H. Wong^3^, Bronwyn H. Bradshaw-Hajek,^2^ Marta Krasowska*,^1,2^ and David A. Beattie*^1,2^

^1^ Future Industries Institute, University of South Australia, Mawson Lakes, South Australia 5095, Australia.

^2^ UniSA STEM, University of South Australia, Mawson Lakes, South Australia 5095, Australia.

^3^ Centre for Advanced Macromolecular Design and Australian Centre for NanoMedicine, School of Chemical Engineering, University of New South Wales, New South Wales 2052, Australia.

^4^ Department of Engineering and Technology of Chemical Processes, Faculty of Chemistry, Wrocław University of Science and Technology, Wybrzeże Wyspiańskiego 27, 50-370 Wrocław, Poland.

† These authors contributed equally as primary authors of this work

* Corresponding Authors (Email: [David.Beattie@unisa.edu.au](mailto:David.Beattie@unisa.edu.au) and Marta.Krasowska@unisa.edu.au)

#### **Supplementary Information for Multilayer formation**

To enable peak assignments to be made for the ATR FTIR spectra, solution spectra of concentrated polymer and peptide solutions were first acquired. The ATR FTIR solution spectra of nisin Z is given in Figure S1. There are two dominant peaks present in the spectra at 1645 cm^–1^ and 1538 cm^–1^, attributable to amide I and amide II ^1^ bands respectively. The peak containing the most information is the amide I peak, which is almost entirely due to the C=O stretch vibrations of the linkage bonds ^2^. The amide I peak is broad owing to the many underlying components. It is the positions of these underlying components that can give an indication of structural composition and conformational changes of the polymer. In contrast, the amide II band derives predominantly from NH bending as well as in part from the CN stretching vibration ^2^ providing much less conformational information than the adjacent amide I peak.


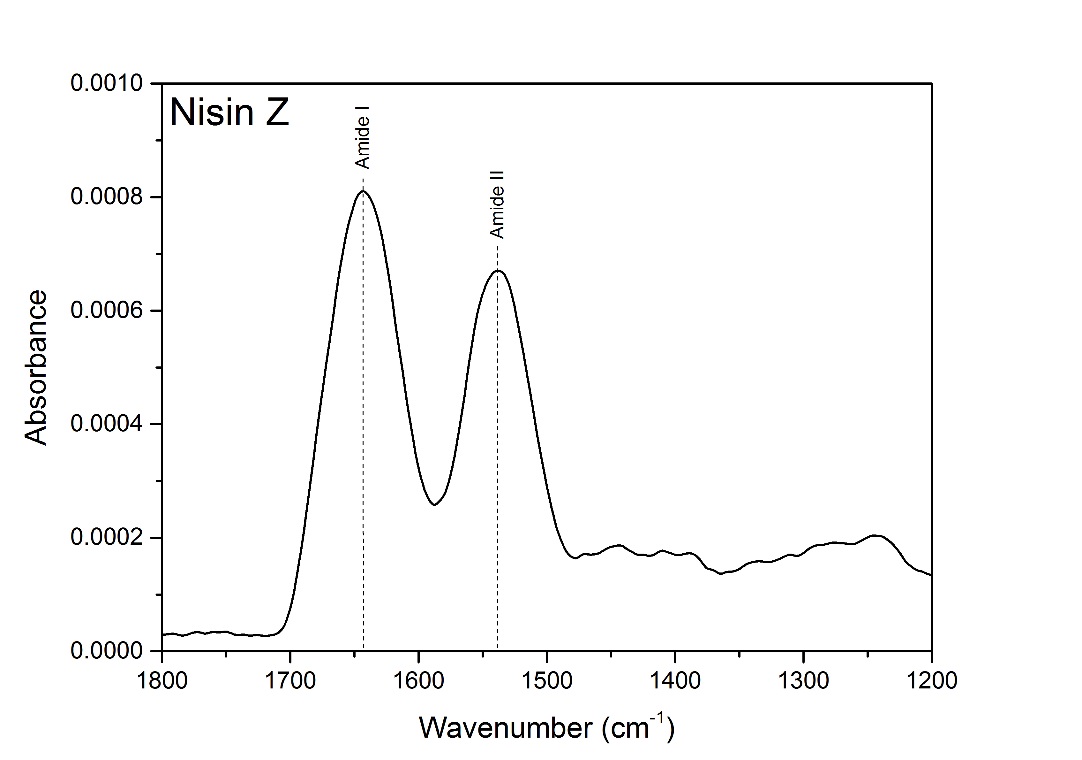


**Figure S1:** Solution ATR FTIR spectrum for a concentrated 10,000 ppm solution of nisin Z in 0.1M KCl at pH 6. Peak assignments are indicated above the major peaks.

Both polysaccharide solution spectra are presented in Figure S2, with respective peak assignments listed in Table S1. The peak assignments for the solution spectra were based on previous work from our research group^3, 4^ and others^1, 5, 6^. The solution spectra of chitosan in Figure S2, panel A contains two large peaks at 1551 cm^–1^ and 1414 cm^–1^. These peaks are, in part, due to the asymmetric and symmetric carboxyl stretch of the acetic acid used to dissolve the chitosan. These large peaks overlap with others that are attributable to chitosan, namely the CH_2_ groups of the polysaccharide, as well as the amide II peak^4^. In addition, the spectra also contain a range of peaks that arise from the glycosidic C-O-C/C-N and C-O-C linkages at 1153 cm^–1^, 1092 cm^–1^ and 1076 cm^–1^ with a final peak at 1020 cm^–1^ due to the skeletal C-O stretch between the chitosan monomers.

The solution spectra of carrageenan presented in Figure S2, panel B contains two main groupings of peaks in the fingerprint region between 1300 cm^–1^ and 800 cm^–1^. Peaks at 1245 cm^–1^ and 1223 cm^–1^ are attributed to the asymmetric stretch of the sulfate groups of carrageenan. Similar to the chitosan spectrum, the spectrum for carrageenan also contains peaks attributable to the glycosidic linkages, specifically, peaks attributable to C-O-C at 1154 cm^–1^, 1064 cm^–1^, 1043 cm^–1^ and 1012 cm^–1^, with a peak attributable again, to skeletal C-O stretching vibrations at 931 cm^–1^. Also present is a peak at 1634 cm^–1^, that may arise not from the carrageenan itself, but from the carboxylate group of potentially present uronic acid^7, 8^.


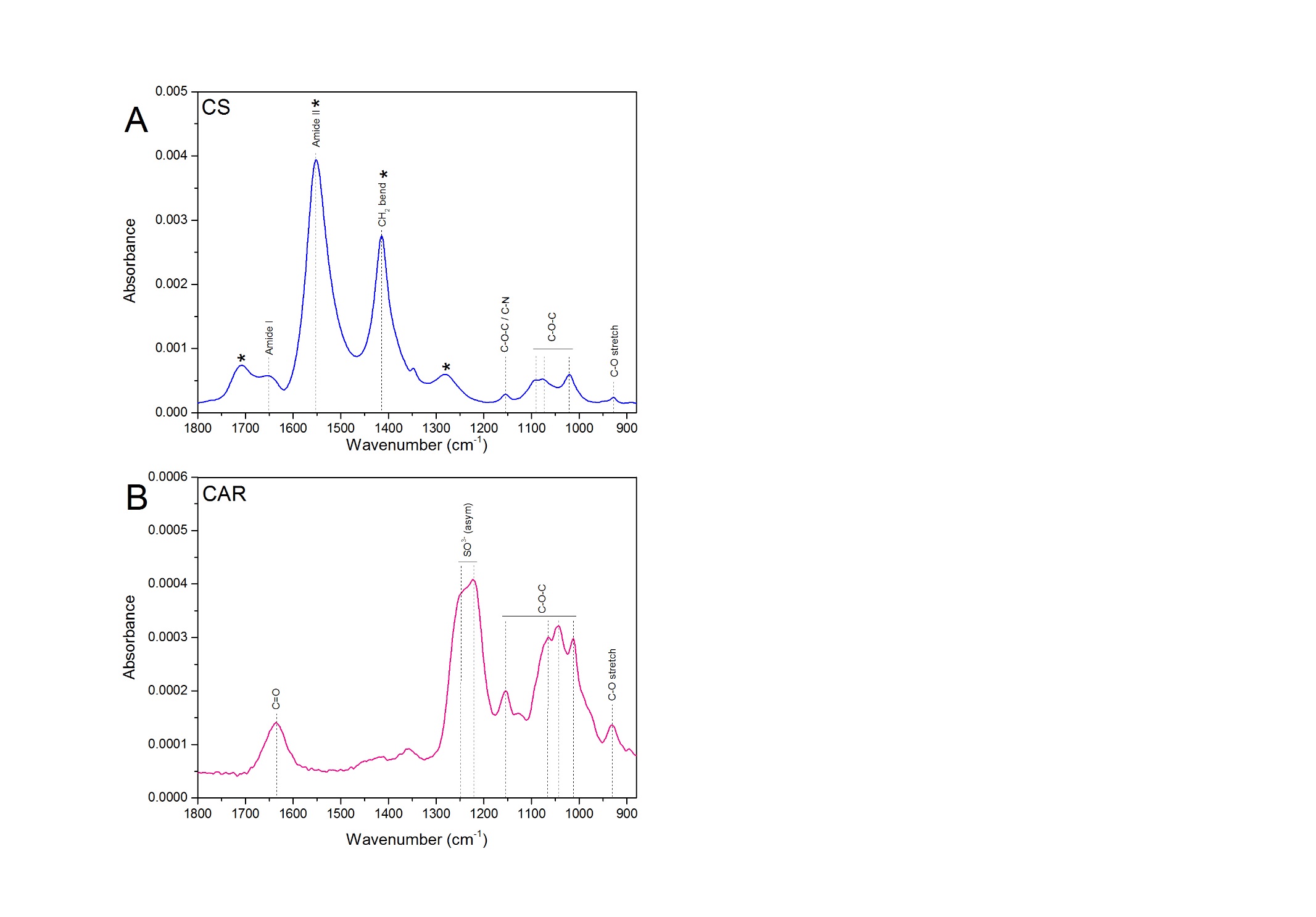


**Figure S2:** Solution ATR FTIR spectrum for concentrated 5000 ppm solutions of CS (panel A) and CAR (panel B) in 0.1 M KCl at pH 6. Full peak assignments are given in Table S1. Asterisks in the spectrum for CS represent peaks that arise from acetic acid, which in some cases overlap other peaks attributed to CS.

**Table S1:** Peak positions and assignments for (i) ATR FTIR spectrum of CAR solution, 6000 mg.L^-1^, pH 6 and 0.1 M KCl; (ii) ATR FTIR spectrum of CS solution, 6000 mg.L^-1^, pH 6 and 0.1 M KCl; and (iii) ATR FTIR spectrum of nisin Z solution, 10,000 mg.L^-1^, pH 6 and 0.1 M KCl.

| Peak Positions (cm^-1^) | | | Peak Assignment |
| --- | --- | --- | --- |
| CAR solution | **CS solution** | **Nisin solution** |  |
|  | 1644 | 1644 | Amide I^1, 3^ |
| 1634^b^ |  |  | ν(C=O), δ(O-H) |
|  | 1551^a^ | 1538 | Amide II^1, 3^ |
|  | 1414 ^a^ |  | δ(CH_2_)^3^ |
|  | 1380 |  | δ(CH_3_)^3^ |
|  | 1279 ^a^ |  | ν(C-O) |
| 1248 |  |  | ν_as_(SO_3_^-^)^5^ |
| 1221 |  |  | ν_as_(SO_3_^-^)^5, 6^ |
| 1154 | 1154 |  | ν(C-O-C),ν(C-N)^3^ |
|  | 1092 |  | ν_as_(C-O-C)^3^ |
|  | 1076 |  | ν_as_(C-O-C)^3^ |
| 1064 |  |  | ν_as_(C-O-C)^6^ |
| 1043 |  |  | ν_as_(C-O-C)^6^ |
|  | 1020 ^a^ |  | ν(C-O)^3^ |
| 1012 |  |  | ν_as_(C-O-C)^6^ |
| 931 | 930 |  | ν(C-O)^6^ |
| 838 |  |  | ν(C-O-S)^3^ |

^a^ Peaks dominated by acetic acid used to dissolve chitosan, ^b^ most likely from uronic acid present in the carrageenan. Annotations: ν – stretching vibrations, ν_s_ – symmetric stretching vibrations, ν_as_ – asymmetric stretching vibrations, δ – in-plane bending vibrations

The mass of the polymers contained in the multilayer can be quantified using the method proposed by Pitt and Cooper^9^. The prominent sulfate peaks between 1248 cm^–1^ and 1219 cm^–1^ were used for the calculations for CAR. Further, the glycosidic linkage peaks between 1160 cm^–1^ and 1000 cm^–1^ were used for the CS calculations. Finally, the amide II band at 1538 cm^–1^ was used for the nisin calculations, as it is the peak that is the least affected by overlap with other peaks and also least affected by the O-H bending region attributable to water. The resulting calibration curves for these three molecules can be seen in Figure S3, where CAR solutions ($0.5-6 mg\cdot\mathrm{mL}^{-1}$) were flowed over the bare ZnSe IRE and CS ($0.5-6 mg\cdot\mathrm{mL}^{-1}$) and nisin Z solutions (0.5 – 10$mg\cdot\mathrm{mL}^{-1}$) were flowed over a layer of PEI adsorbed onto the ZnSe IRE. The extracted parameters required for the calculations are presented in Table S2. Using these parameters, and spectra for each individually adsorbed layer, the growth of the multilayer can be quantitatively determined.


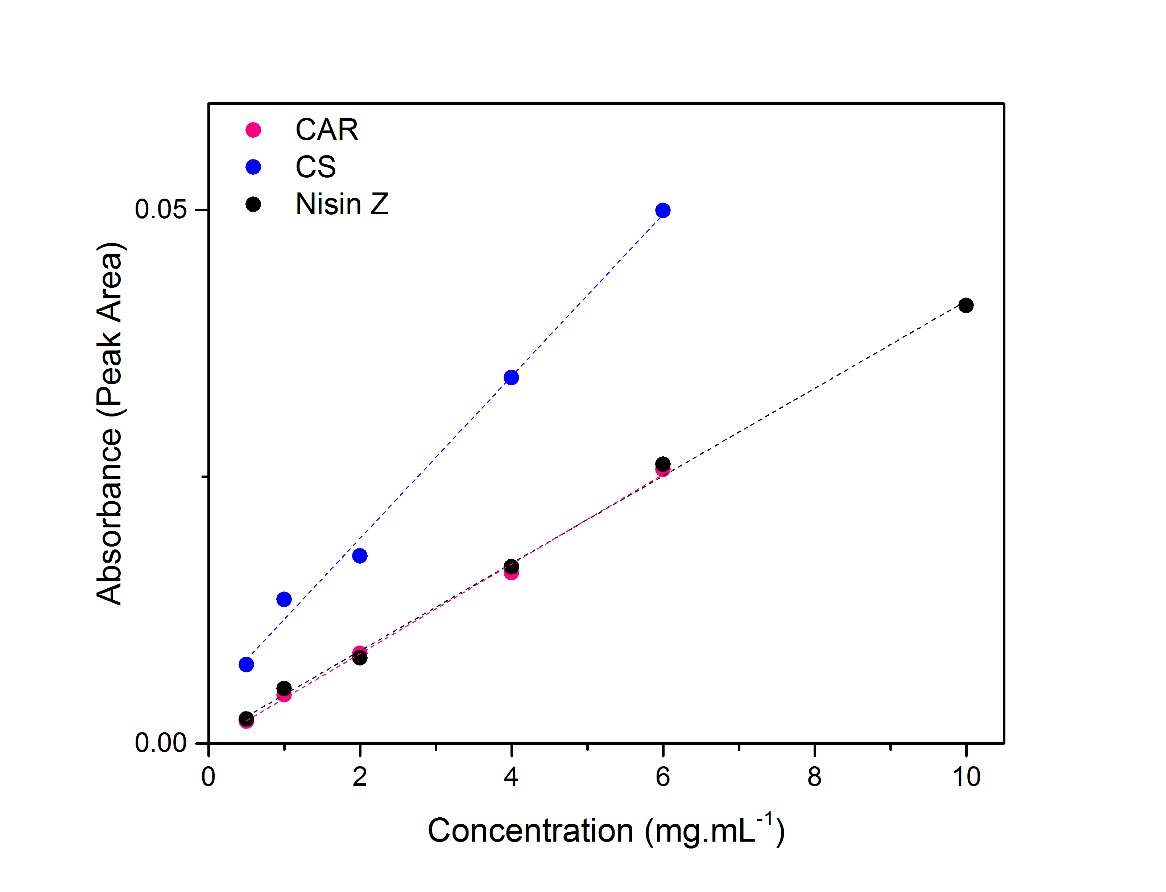


**Figure S3:** Calibration curves for CAR, CS and nisin Z, where CAR solutions ($0.5 - 6 mg\cdot\mathrm{mL}^{-1}$) were flowed over the bare ZnSe IRE and CS ($0.5 - 6 mg\cdot\mathrm{mL}^{-1}$) and nisin Z solutions ($0.5 - 10 mg\cdot\mathrm{mL}^{-1}$) were flowed over a layer of PEI adsorbed onto the ZnSe IRE.

**Table S2:** Characteristic peaks, peaks position, penetration depth and calibration constant, K for CAR, CS and nisin Z.

| Characteristic Peak | Position $\boldsymbol{(}\boldsymbol{cm}^{\boldsymbol{-1}}\boldsymbol{)}$ | Penetration Depth $\mathbf{(cm}\boldsymbol{)}$ | K abs$\boldsymbol{(}\mathbf{ml}\mathbf{mg)}^{\mathbf{-1}}$ |
| --- | --- | --- | --- |
| CAR (average) | 1233 | $1.21\times{10}^{-4}$ | $4.13\times{10}^{-3}$ |
| CS (average) | 1080 | $1.38\times{10}^{-4}$ | $7.95\times{10}^{-3}$ |
| Nisin Z | 1538 | $9.71\times{10}^{-5}$ | $4.08\times{10}^{-3}$ |

#### **Supplementary Information for Multilayer Characterization**


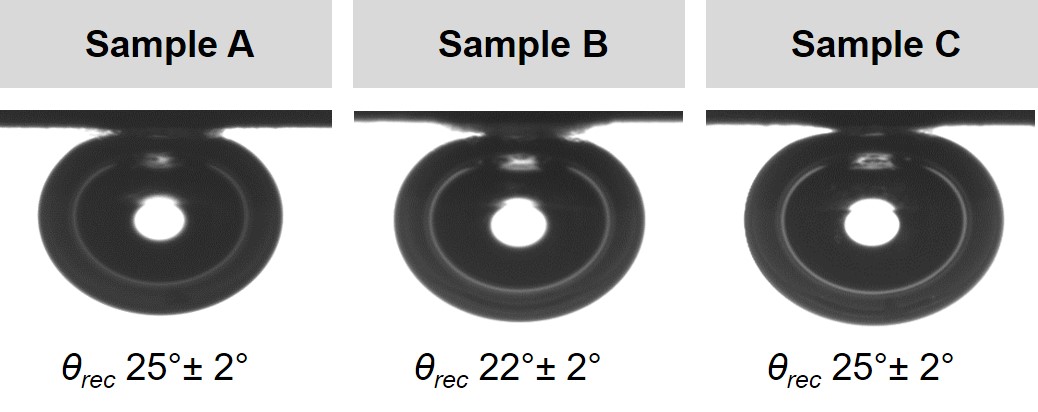


**Figure S4:** Receding water contact angles of Samples A, B and C determined using AFM. Sample A: PEM with nisin Z outer layer; Sample B: PEM with nisin Z/CAR outer layer and; Sample C: PEM without nisin.

**
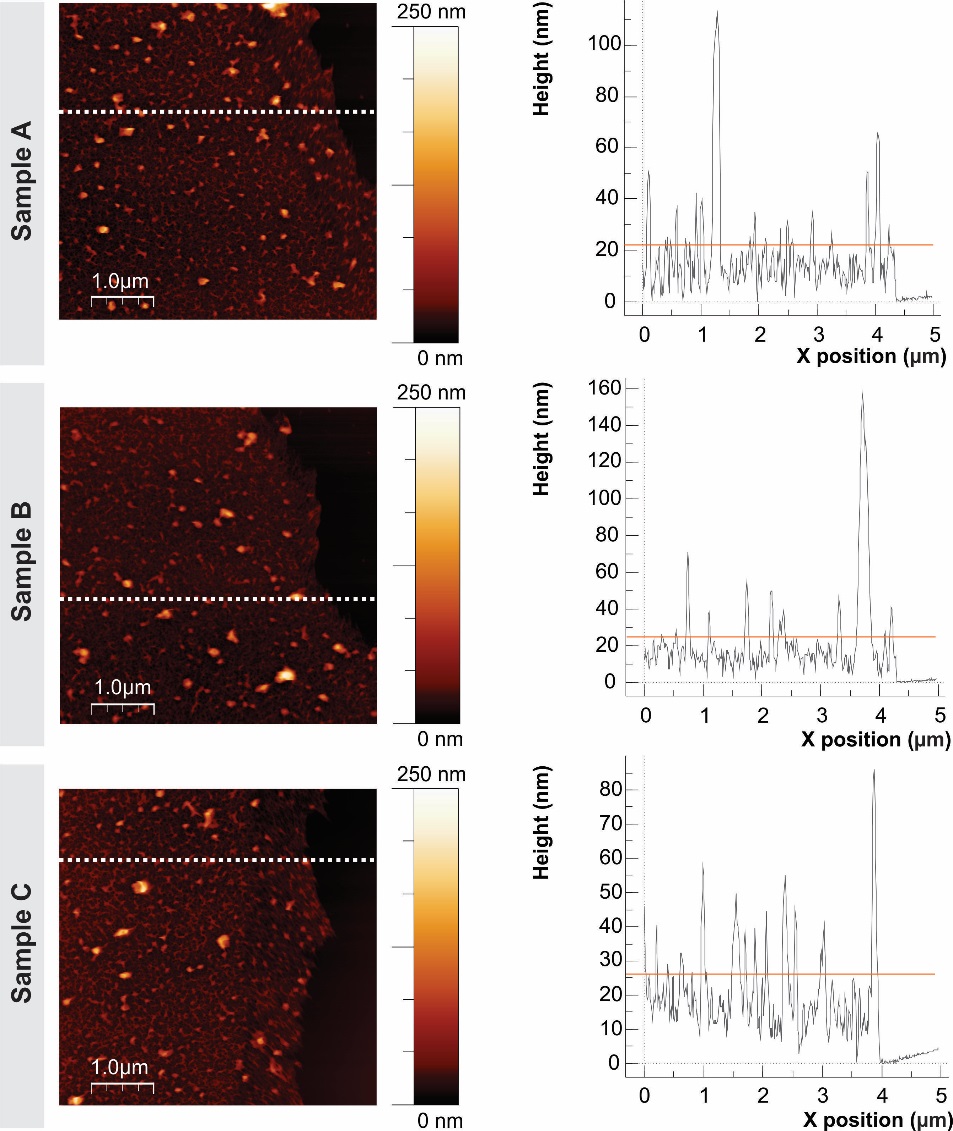
**

**Figure S5:** AFM 5 × 5 μm^2^ topography images collected across the scratch and in 0.1 M KCl for Samples A, B and C are displayed on the left. The cross section of the relevant images (across the dashed white lines) are presented on the right. Sample A: PEM with nisin Z outer layer; Sample B: PEM with nisin Z/CAR outer layer and; Sample C: PEM without nisin Z.


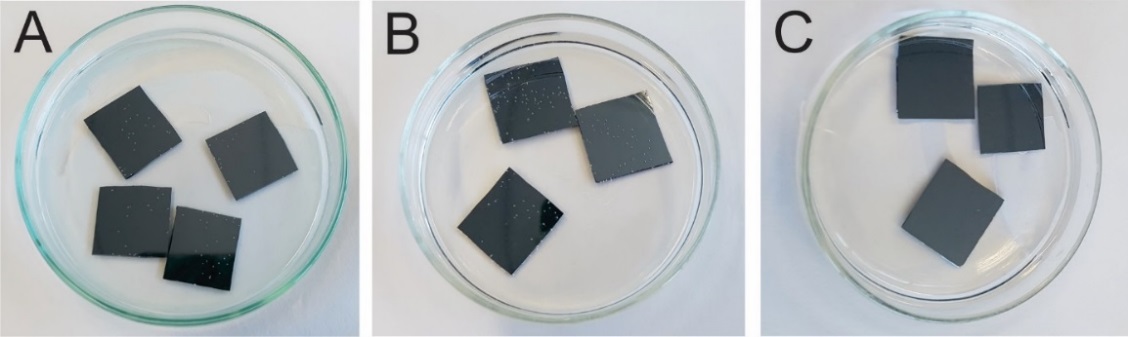


**Figure S6:** Photographs of sample types A, B and C observed after being transferred from the fridge (4 °C) to T = 22 °C. Samples A: PEM with nisin Z outer layer; Samples B: PEM with nisin Z/CAR outer layer and; Samples C: PEM without nisin Z. Nucleated gas can be observed on samples A and B upon return to ambient room temperature, while samples C present no observable gas bubbles.

#### **Supplementary Information for Antimicrobial Activity**

**Table S3:** Viability of S. aureus and MRSA planktonic cells in the presence of the glass slides. Sample A: PEM with nisin outer layer; Sample B: PEM with nisin/CAR outer layer; Sample C: PEM without nisin and; Sample D: Blank glass slide. The data are a minimum of two biological replicates.

| **Sample** | log_10_Planktonic/CFU.mL^-1^ | |
| --- | --- | --- |
|  | ***S. aureus*** | **MRSA** |
| **A** | 6.92 ± 0.17 | 4.47 ± 0.57 |
| **B** | 6.62 ± 0.16 | 4.48 ± 0.26 |
| **C** | 8.24 ± 0.16 | 7.95 ± 0.06 |
| **D** | 8.08 ± 0.32 | 8.19 ± 0.17 |

**Table S4:** Viability of S. aureus and MRSA biofilm cells in the presence of glass slides. Sample A: PEM with nisin Z outer layer; Sample B: PEM with nisin Z/CAR outer layer; Sample C: PEM without nisin Z and; Sample D: Blank glass slide. The data are a minimum of two biological replicates.

| **Sample** | log_10_Biofilm/CFU.cm^-2^ | |
| --- | --- | --- |
|  | ***S. aureus*** | **MRSA** |
| **A** | 5.24 ± 0.16 | 2.92 ± 0.16 |
| **B** | 4.13 ± 0.16 | 3.05 ± 0.16 |
| **C** | 8.47 ± 0.16 | 8.19 ± 0.16 |
| **D** | 8.37 ± 0.16 | 7.98 ± 0.16 |


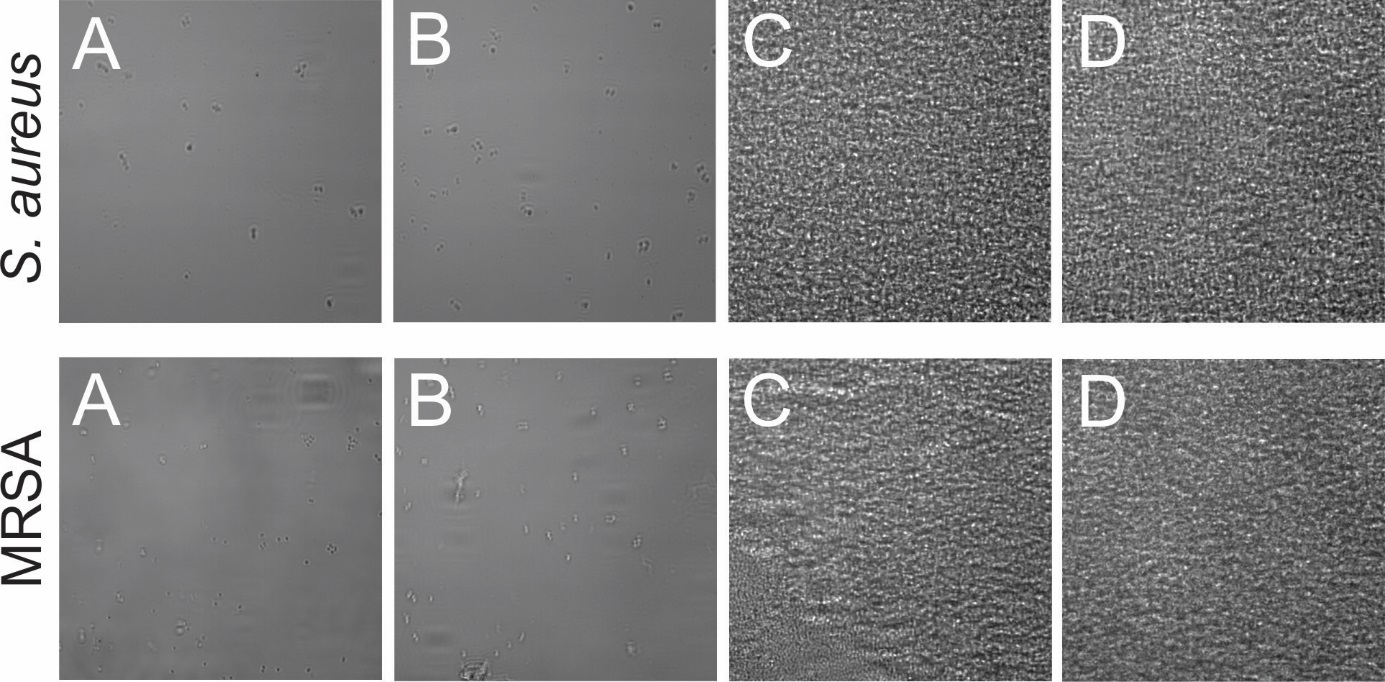


**Figure S7:** Light microscope images of glass slides that were exposed to S. aureus (top) and MRSA (bottom) bacteria culture for 6.5 h. Sample A: PEM with nisin Z outer layer; Sample B: PEM with nisin Z/CAR outer layer; Sample C: PEM without nisin Z and; Sample D: Blank glass slide.

#### **Supplementary Information for Materials and Methods**


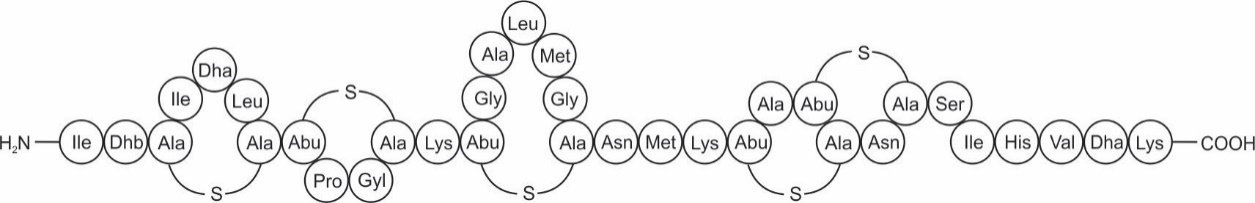


**Figure S8**: The structure of the peptide Nisin Z.


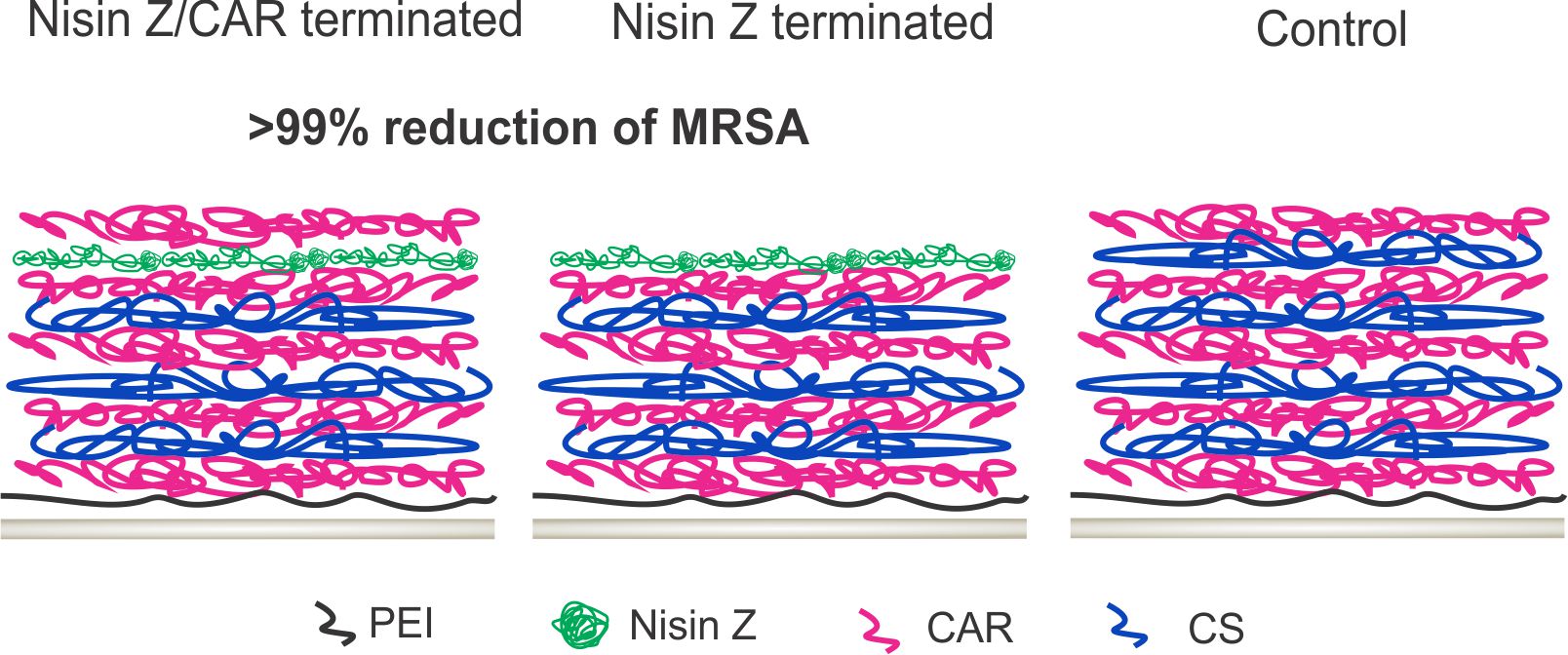


**Figure S9**: Schematic diagram of the multilayer formation architecture.

**REFERENCES**

1. A. Barth, *Biochimica et Biophysica Acta (BBA) - Bioenergetics*, 2007, **1767**, 1073-1101.

2. J. Kong and S. Yu, *Acta Biochimica et Biophysica Sinica*, 2007, **39**, 549-559.

3. N. L. Benbow, J. L. Webber, S. Karpiniec, M. Krasowska, J. K. Ferri and D. A. Beattie, *Physical Chemistry Chemical Physics*, 2017, **19**, 23790-23801.

4. T. T. M. Ho, K. E. Bremmell, M. Krasowska, S. V. MacWilliams, C. J. E. Richard, D. N. Stringer and D. A. Beattie, *Langmuir*, 2015, **31**, 11249-11259.

5. B. Matsuhiro, *Hydrobiologia*, 1996, **326**, 481-489.

6. E. Brychcy, M. Malik, P. Drożdżewski, Ż. Król and A. Jarmoluk, *Polymers*, 2015, **7**, 2638-2649.

7. V. A. Cosenza, D. A. Navarro and C. A. Stortz, *Carbohydrate Polymers*, 2017, **157**, 156-166.

8. M. Arman and S. A. U. Qader, *Carbohydrate Polymers*, 2012, **88**, 1264-1271.

9. W. G. Pitt and S. L. Cooper, *Journal of Biomedical Materials Research*, 1988, **22**, 359-382.
